# Exploring Multifaceted Roles of Bambusicolous *Apiospora* in *Phyllostachys bambusoides*

**DOI:** 10.1007/s00248-025-02631-z

**Published:** 2025-11-05

**Authors:** Sun Lul Kwon, Chang Wan Seo, Haeun Kwon, Minseo Cho, Yeonjae Yoo, Sang Hyun Lee, Dae Young Kwon, Young Min Lee, Young Mok Heo, Gyu Hyeok Kim, Young Woon Lim, Dongho Lee, Yong-Seok Choi, Hanbyul Lee, Jae-Jin Kim

**Affiliations:** 1 Division of Environmental Science & Ecological Engineering, College of Life Science & Biotechnology, Korea University, Seoul, Republic of Korea; 2 School of Biological Sciences and Institute of Biodiversity, Seoul National University, Seoul, Republic of Korea; 3 Department of Plant Biotechnology, College of Life Sciences and Biotechnology, Korea University, Seoul, Republic of Korea; 4 R&I Center, COSMAX BTI, Seongnam, Republic of Korea; 5 Division of Wood Engineering, Forest Products and Industry Department, National Institute of Forest Science, Seoul, Republic of Korea; 6 Division of Life Sciences, Korea Polar Research Institute, Incheon, Republic of Korea

**Keywords:** Amplicon analysis, *Apiospora hysterina*, Bamboo culm, Endophytic fungi, Plant hormone, Plant pathogen

## Abstract

Bamboo plays a crucial role in mitigating climate change. Among various microorganisms inhabiting bamboo, *Apiospora* is a common bambusicolous fungus that induces black spots, functioning either as a saprobe or as a plant pathogen. However, the diversity and ecological roles of *Apiospora* as an endophyte in bamboo remain poorly understood. This study explored the diversity and ecological functions of bambusicolous *Apiospora* in *Phyllostachys bambusoides* forests. Bamboo samples representing different stages—young (1-year-old, without black spots), mature (aged 3 years, few black spots), and dead (with many black spots)—were collected. Mycobiome analyses across different tissues (culm, leaf, root) and environmental samples (forest soil) revealed diverse *Apiospora* species throughout the bamboo lifecycle. Notably, *Apiospora hysterina* emerged as a prevalent endophyte, inhabiting not only mature but also younger, healthier bamboo stages. Biological activity assays, including antioxidant, antifungal, and plant hormone tests, indicated that *A. hysterina* exhibits potential mutualistic interactions beneficial to bamboo. Conversely, genomic analyses of carbohydrate-active enzyme profiles, effector/virulence factors, and putative biosynthetic gene clusters suggested potential pathogenic capabilities that may involve secondary metabolites, though functional validation is required. These findings reveal the widespread presence of *Apiospora* species as endophytes from the early to senescent bamboo stages, highlighting *A. hysterina*’s dual capacity as a symbiont and pathogen. Our study underscores the complexity of bambusicolous *Apiospora*’s ecological roles, emphasizing the need for further investigation into its interactions with bamboo ecosystems.

**Supplementary Information:**

The online version contains supplementary material available at 10.1007/s00248-025-02631-z.

## Introduction

Bamboo (family Poaceae, subfamily Bambusoideae) is a valuable grass plant renowned for diverse ecological roles, including biodiversity conservation [1], carbon fixation [2], soil and water conservation [3], and economic activities [4]. *Phyllostachys*, known as running bamboo, is one of the largest bamboo genera, consisting of approximately 50 species, predominantly found in Asia (China and Japan). In Korea, bamboo forests cover an area of approximately 22,000 ha, mainly consisting of *Phyllostachys* species (*P. bambusoides*, *P. nigra* var. *henonis*, and *P. edulis*), with 96% located in the southern region [5]. Bamboo has a unique growth cycle consisting of rapid growth, maturation, and senescence. During the growth phase, culms quickly elongate, achieving full height while relying on rhizome reserves, with minimal lignification but significant biomass accumulation [6, 7]. This stage allows for colonization by endophytes, potentially enhancing growth and stress tolerance [8]. In maturation, vertical growth stops, and resources shift to lignification, leaf and branch development, and carbon storage, ensuring structural stability [9–12]. Aging tissues during this stage can become more vulnerable to pathogens, resulting in foliar spots or culm deterioration [13]. In the senescent stage, dead culms, which are increasingly lignified and silica-rich, decompose slowly and serve as important substrates for saprobic fungi, aiding nutrient cycling and ecosystem renewal [10, 14–16].

Bambusicolous fungi live on various bamboo substrates, including culms, leaves, branches, rhizomes, and roots, and play a crucial role in bamboo diversity conservation and ecosystem maintenance [14]. Over 1100 species within 228 genera have been identified as bambusicolous fungi, primarily including ascomycetes (630 species), basidiomycetes (150 species), and anamorphic fungi (330 species) [17, 18]. These fungi significantly influence bamboo ecosystems by functioning as endophytes, pathogens, and saprobes.

Traditionally, fungal endophytes were defined as fungi residing within plant tissues without causing visible disease symptoms [8]. Based on this definition, endophyte-plant symbiosis provides valuable insights into plant–microbe interactions with potential applications in agriculture and environmental sustainability [19, 20]. For instance, fungal endophytes can produce growth hormones, such as gibberellins and auxins, promoting plant growth, enhancing root development, and improving overall plant health [19]. Bambusicolous endophytic fungi are known to synthesize bioactive compounds with antioxidants, antimicrobial, antitumor, and plant growth–promoting activities [21]. Beyond these functional benefits, the definition of endophytes has been refined over time. More recent perspectives emphasize their dynamic ecological roles, which may shift along a continuum from mutualism to commensalism or even pathogenicity depending on the host’s developmental stage, physiology, and environmental conditions [13, 22]. This perspective indicates that endophytes should not be regarded solely as beneficial symbionts but rather as versatile members of the plant microbiome, whose interactions with their hosts are inherently context dependent. In line with this perspective, endophytes are increasingly being redefined not by a narrow functional characterization but as a broad concept encompassing all fungi inhabiting the internal tissues of their hosts.

Among bambusicolous fungi, the genus *Apiospora* is particularly noteworthy. According to the Index Fungorum (2024), 175 epithets of *Apiospora*, with 42 species, are recorded as bambusicolous fungi. Many *Apiospora* species are saprobes, causing black spot formation on dead or decaying bamboo [23, 24], and some are pathogenic [25–27]. Additionally, *Apiospora* species are prevalent as endophytes in running bamboo (*Phyllostachys* spp.) and dwarf bamboo (*Sasa* spp.) in Japan [28], offering competitive advantages to their hosts through hormone synthesis, seed germination promotion, and antimicrobial and antioxidant activities [29]. *Apiospora* has also been reported from healthy bamboo leaves and isolated from bamboo shoots [30, 31]. These findings underscore the ecological versatility of *Apiospora* as saprobes, pathogens, and endophytes [18, 28, 32].

Thus, we hypothesize that bambusicolous *Apiospora* species significantly influence the bamboo lifecycle, serving as symbionts during growth stages, pathogens during maturation, and saprobes in dead bamboo tissues. However, the diversity, ecological traits, and symbiotic interactions of endophytic *Apiospora* in bamboo hosts remain unclear. This study aimed to explore the diversity of bamboo endophytic fungi, focusing on *Apiospora*, across various bamboo tissues (culm, leaf, root) and soil, as well as different bamboo stages (young, mature, dead). We investigated the ecological preferences (niches) of these endophytes and analyzed the biological activities and genetic characteristics, including trophic lifestyle prediction using CAZyme and effector/virulence factor profiling, to elucidate their multifaceted ecological roles in Korean *Phyllostachys* forests.

## Methods

Detailed descriptions are provided in the Supplementary Text.

### Sampling Collection and Treatment

Bamboo materials (*Phyllostachys bambusoides*) were collected during the winter season (in December 2021) in the bamboo forest of Juknokwon, Damyang-gun, Jeollanam-do, Korea. The Juknokwon, located at 35.3281°N, 126.9858°E, is a 160,000 m^2^ bamboo forest park set in a mixed landscape of mountains and plains (Additional File 1: Fig. S1). The region has a humid subtropical climate with hot, rainy summers (22 to 30 °C) and cold, dry winters (− 5 to 4 °C). The bamboo materials were classified into three stages: “young,” green, healthy, without black spots and wounds, fast growing, and aged less than a year; “mature,” green, with few black spots and wounds, slow growing, and aged 3 years; and “dead,” yellowish brown, with many black spots and wounds, dried, and aged more than 5 years. Each bamboo material was divided into three tissue types (culm, leaf, and root). The bamboo forest soils were collected on the same day and location. All samples were stored at − 20 °C before DNA extraction and then transferred to a − 80 °C deep freezer for long-term storage. To prevent disinfectants from penetrating internal tissues through wounds, only undamaged parts of bamboo tissues were selected for endophytic fungal analysis. Upon transporting the samples to the laboratory, surface sterilization was performed following the methodology of Barra et al. [33]. To verify that surface sterilization was effective in eliminating external infectant, sterilized bamboo tissue’s surfaces were imprinted onto culture media, and the absence of fungal growth was confirmed. The samples were then crushed in liquid nitrogen using a Freezer Mill (SPEX 6875D).

### Bambusicolous Fungal Community Analysis

DNA was extracted using DNeasy PowerLyzer PowerSoil Kit (QIAGEN). Polymerase chain reaction amplified ITS2 regions with primers ITS3 and ITS4. Libraries were sequenced on Illumina MiSeq (2 × 300 bp, paired end). ITS2 sequences were analyzed using QIIME2. DADA2 plugin filtered low-quality, short, and chimeric sequences. Operational taxonomic units (OTUs) were clustered at 99% similarity using VSEARCH, excluding OTUs < 10 sequences. Taxonomy assignments utilized modified UNITE v8.3 and enhanced GenBank sequences. Phylogenetic analysis used MAFFT and RAxML with reference sequences of *Apiospora* and related genera from the GenBank database (Additional File 2: Table S1). Community variation was assessed using α- and β-diversity analyses implemented via the “vegan” package. Principal coordinate analysis (PCoA), PERMANOVA (including pairwise tests) with Bray–Curtis dissimilarities, β-dispersion, β-diversity partitioning (turnover, nestedness, total), and dbRDA were applied to identify explanatory factors and interpret community variation. PCoA ordination plot, Venn diagrams, linear discriminant analysis (LEfSe), and heatmaps (“pheatmap”) were used to visualize data. Indicator species were analyzed with the “Indicspecies” package. The trophic modes were assigned using FUNGuild.

### Biological Activity and Metabolite Analysis

Nine strains from the Korea University Culture Collection, representing three species (*Apiospora arundinis*, *Apiospora camelliae-sinensis*, *Apiospora hysterina*), were analyzed based on mycobiome findings. Extracts were prepared from strains cultured on potato dextrose agar, extracted with methanol and ethyl acetate. Radical scavenging activities were measured using 2,2′-azino-bis(3-ethylbenzothiazoline-6-sulfonic acid) (ABTS) and 2,2-diphenyl-1-picrylhydrazyl (DPPH) assays with standard controls (Trolox and l-ascorbic acid, respectively). Antifungal activity against *Botrytis cinerea*, *Colletotrichum gloeosporioides*, and *Fusarium oxysporum* was assessed via disk diffusion assay. Indole-3-acetic acid (IAA), abscisic acid (ABA), and gibberellic acid (GA) production by strains was evaluated using liquid chromatography with tandem mass spectrometry (LC–MS/MS) with an Orbitrap Exploris 120 mass spectrometer. Molecular networks were generated using GNPS and visualized in Cytoscape. Additionally, MolNetEnhancer was applied to further improve the annotation of the molecular networks and support the prediction of major chemical classes. A direct link to the molecular network can be accessed (https://gnps.ucsd.edu/ProteoSAFe/status.jsp?task=380a4dc036a54fcb9870206736a59901).

### Whole Genome Analysis

Genomic DNA from selected *Apiospora* was sequenced with the PacBio Sequel and NovaSeq 6000 platforms. Assembly involved Flye, Racon, and Hapo-G. Genomes were annotated with RepeatModeler, RepeatMasker, BRAKER2, GeMoMa, tRNAscan-SE, Rfam, and MMseqs2. CAZyme-related genes were identified via dbCAN3, with trophic lifestyles classified by CATAStrophy. Secondary metabolite clusters identified using antiSMASH. antiSMASH similarity scores represent the percentage of genes in reference clusters with sequence matches to query regions based on BLAST scoring, rather than comprehensive functional similarity assessments. Genome-scale modeling and BLAST analyses identified hormone synthesis pathways, visualized using ChemDraw.

### Trophic Lifestyle Classification With Genomic Profiles

To predict the trophic lifestyles of the bambusicolous *Apiospora* species, CATAStrophy (version 0.1.0) and Predector (version 1.2.7) were applied. For CATAStrophy analysis, CAZyme-related genes were first annotated using dbCAN3 with HMMER-based domain searches and streamlined to the CATAStrophy pipeline to classify fungal trophic classes based on default reference datasets. Principal component analysis (PCA) was conducted to visualize and compare the trophic strategies of *Apiospora* strains against reference species. Predector analysis was performed with the complete proteome to predict effector genes, secretion systems, and virulence factors, with results compared with data from previous research [34].

## Results

### Fungal Mycobiome Analysis

#### Fungal Mycobiomes of Bamboo and Soil Samples

A total of 89 bamboo forest samples (26 culms, 27 leaves, 27 roots, and 9 soil types) were collected for fungal mycobiome analysis. For ITS2 amplicon analysis, 14,439,126 paired sequences were obtained, and 9,205,658 merged paired reads (average length 319 bp) were retained after quality filtering. Rarefaction curves confirmed sufficient sequencing depth (Additional File 1: Fig. S2). After clustering and discarding low coverage mOTUs, 4303 mOTUs were retained. Among these, 31 mOTUs were assigned to the candidate *Apiospora* species. Phylogenetic analysis for precise species identification (Additional File 1: Fig. S3 and Additional File 2: Table S2) revealed 31 mOTUs as seven known *Apiospora* species (*A. arundinis*, *A. hysterina*, *A. camelliae-sinensis*,* A. minutispora*, *A. pseudohyphopodii*, *A. rasikravindrae*, and *Arthrinium phaeospermum*), as well as a new candidate species (*Apiospora* sp. 1) and four indistinct species (ITS_Apio_01, ITS_Apio_12, ITS_Apio_13, and ITS_Apio_29). These 12 *Apiospora* species were used for ALC analysis.

**Fig. 1:**
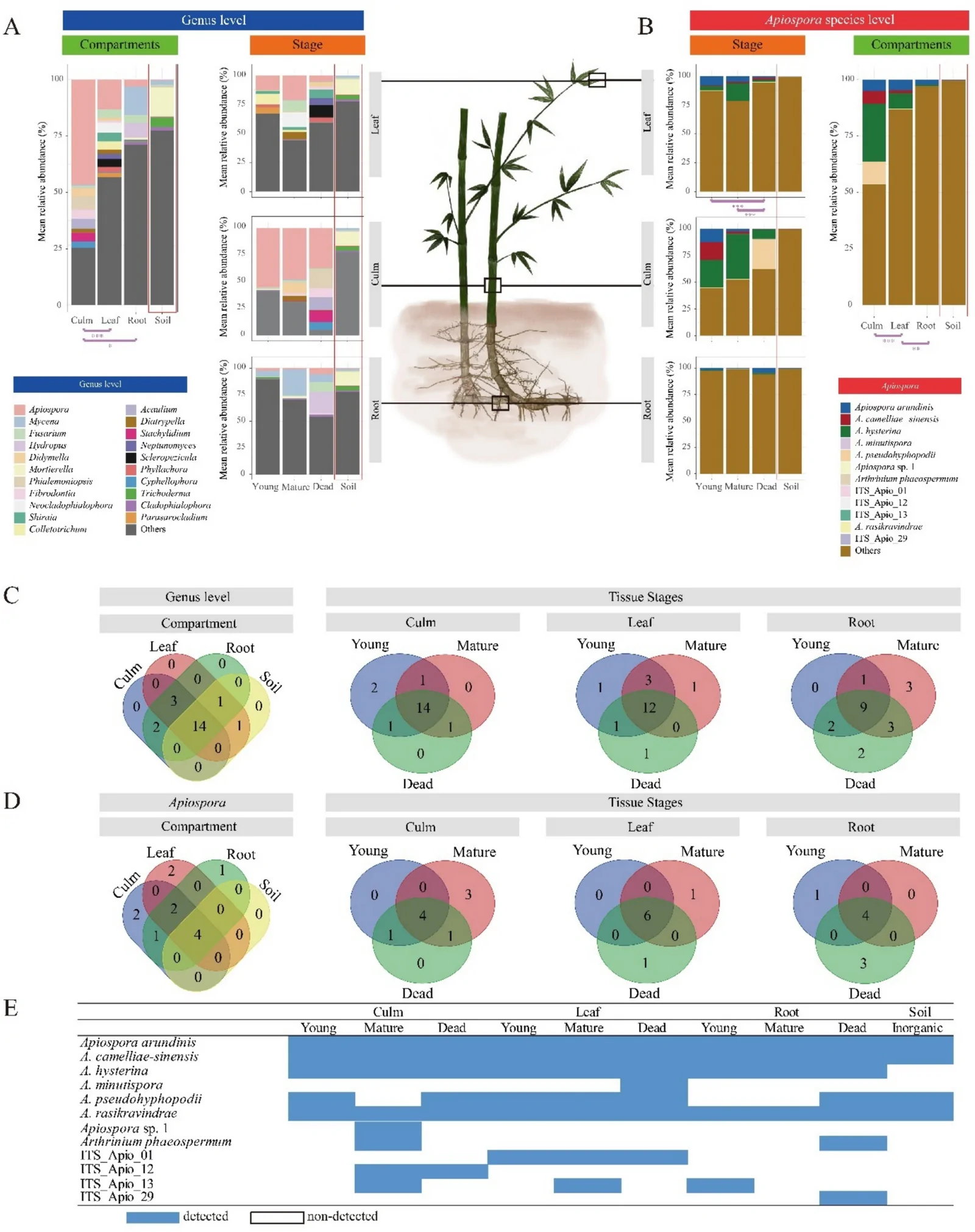
Bambusicolous endophytic fungal community structures. Fungal community structures at the **A** genus level and **B** analysis of *Apiospora* species communities according to factors based on mean relative abundance. Shannon diversity of the communities was compared between the bamboo compartment and each tissue stage. Venn diagram of the genus- and species-level community in **C** and **D**, respectively, showing the overlap frequency between bamboo compartments and between the bamboo tissue stages. **E** Detected or undetected *Apiospora* species across the bamboo compartments and stages are shown in the table. The detected *Apiospora* species are highlighted in blue boxes

For GLC analysis, the 21 most abundant fungal genera (> 0.5% of the total reads) were selected, with the exception of unidentified genera. Among them, *Apiospora* was the most dominant genus in the culms and leaves, with mean relative abundances of 46.56% and 13.25%, respectively (Fig. 1A; Additional File 2: Table S3). Moreover, *Apiospora* was the most dominant genus across the bamboo stages, with high mean relative abundances (young, 22.21%; mature, 23.39%; and dead, 16.33%), but the proportion of *Apiospora* decreased in the dead samples and was minimized in the soil samples (0.37%). In bamboo roots and soils, *Mycena* (12.65%) and *Mortierella* (Mortierellaceae) (12.96%) were the most abundant genera, respectively (Fig. 1A; Additional File 2: Table S3). Among the 21 major genera, 14 were detected in all bamboo compartments (culms, leaves, roots, and soil) (Fig. 1C). In contrast, no unique genera were detected in each bamboo compartment. Across the bamboo tissue stages, 14/19, 12/19, and 9/20 genera were shared between the culm, leaf, and root stages, respectively. The genera detected at all stages in all bamboo tissues were *Apiospora*, *Mycena*, *Fusarium*, *Colletotrichum*, *Neptunomyces*, and *Trichoderma* (Additional File 2: Table S4).

In ALC analysis, four species were commonly detected throughout the life of bamboo tissues: *A. arundinis*, *A. camelliae-sinensis*,* A. hysterina*, and *A. rasikravindrae* (Fig. 1D, E; Additional File 2: Table S4). *A. arundinis*, *A. camelliae-sinensis*, and *A. rasikravindrae* were also detected in soil samples, but *A. hysterina* was not detected in soil samples exhibiting an obligatory association with bamboo.

*A. hysterina* was the most abundant species in young culms (25.9%), mature culms (39.1%), and mature leaves (15.2%) (Fig. 1B). *A. arundinis* was highly abundant in young culms (12.5%) and leaves (8.1%) but was present at a considerably lower proportion in the mature and dead stages. *A. camelliae-sinensis* and *A. pseudohyphopodii* were highly abundant in bamboo culms, followed by *A. hysterina* (Fig. 1B); however, they were only abundant in young and dead culms, respectively (Fig. 1B). *Apiospora* sp. 1 was uniquely detected in mature culm tissues, whereas ITS_Apio_01 was detected at all stages of bamboo leaves growth. *A. minutispora* and ITS_Apio_29 were only detected in dead bamboo tissues (Fig. 1D, E; Additional File 2: Table S4). *Arthrinium phaeospermum* was detected in mature culm and dead root tissues (Fig. 1D, E; Additional File 2: Table S4).

#### Variation in Endophytic Fungal Community and Its Ecological Niche

**Fig. 2:**
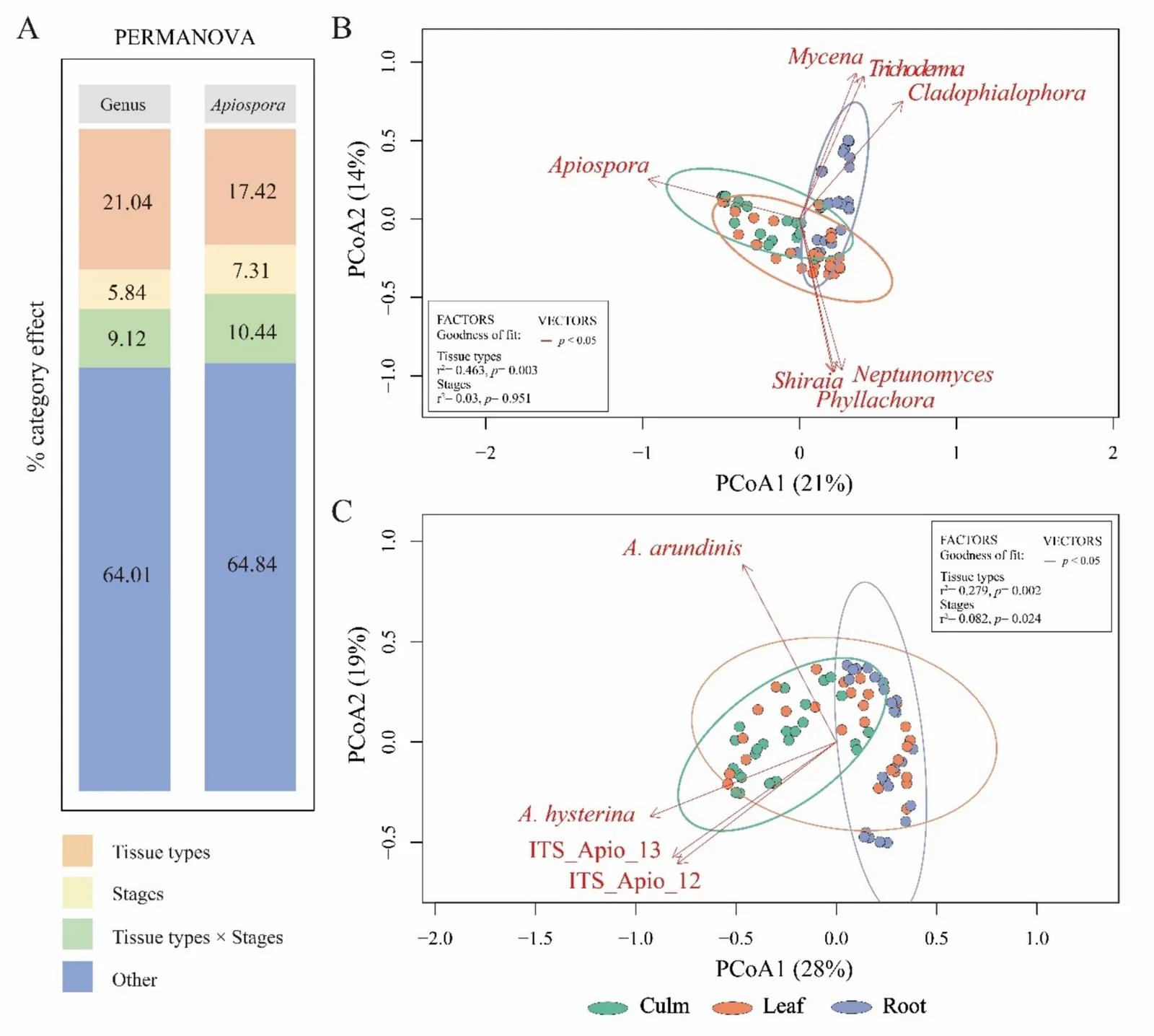
Beta diversity of bambusicolous endophytic fungi. **A** PERMANOVA test was used to determine the effect size of the factors on fungal communities, displaying the percent variation explained by the factors of bamboo “tissue type” (culm, leaf, and root), “stages” (young, mature, and dead), and “combination of both factors.” The undefined effects are summarized under “other.” Principal coordinate analysis ordination plot based on Bray–Curtis distance was used to explore variations in bambusicolous fungal diversity according to the factors. The ellipses indicate clusters of fungal communities according to tissue type. The red arrows indicate significant vectors. The first two principal coordinates explain **B** 35% of the total variance of the genus scale community and **C** 47% of the total variance of the *Apiospora* community. Significance levels are indicated by asterisks (*p < 0.05; **p < 0.01; ***p < 0.001; ns: not significant)

Both α- and β-diversity analyses showed significant differences in endophytic communities across bamboo tissue types (Figs. 1A, B and 2 B, C; Additional File 2: Tables S5 and S6). Within each tissue type, community variation by stage showed significant α-diversity differences only in leaf ALC (Fig. 1B). For β-diversity, significant differences by stage within each tissue were observed in culm and root ALC, as well as leaf GLC, based on PCoA (Additional File 2: Table S6). Differences among bamboo stages were significant only in β-diversity analyses (ALC, Fig. 2C).

PERMANOVA confirmed tissue type as the most influential factor shaping fungal community structure, followed by the combination of tissue type and stage, and stage alone (Fig. 2A). Consistently, dbRDA identified tissue type as the strongest explanatory factor, followed by the combination of tissue stage and stage (Additional File 2: Table S8). Pairwise comparisons revealed no significant differences between young and mature stages within the same tissues, whereas clear dissimilarities were consistently observed between dead and living tissues in both datasets (Additional File 2: Table S7). This indicates that tissue vitality (living vs. dead) exerts a stronger influence on community variation than bamboo stage.

β-Dispersion analysis revealed significant heterogeneity by tissue type at the GLC and by stage at the ALC (Additional File 2: Table S9), suggesting that while PERMANOVA primarily reflects centroid separation among groups, part of the variation could also be attributed to differences in within-group dispersion. Partitioning of Sørensen-based β-diversity demonstrated that overall dissimilarity was dominated by turnover (0.927 at the GLC; 0.827 for ALC), while nestedness contributed more at the ALC (0.087) than at the GLC (0.024) (Additional File 2: Table S10). Notably, turnover was particularly high in culm stages, especially between mature and dead culms (0.35), indicating that specific *Apiospora* species varied substantially across culm stage transitions, particularly reflecting shifts in vitality (Additional File 1: Fig. S4).

**Fig. 3:**
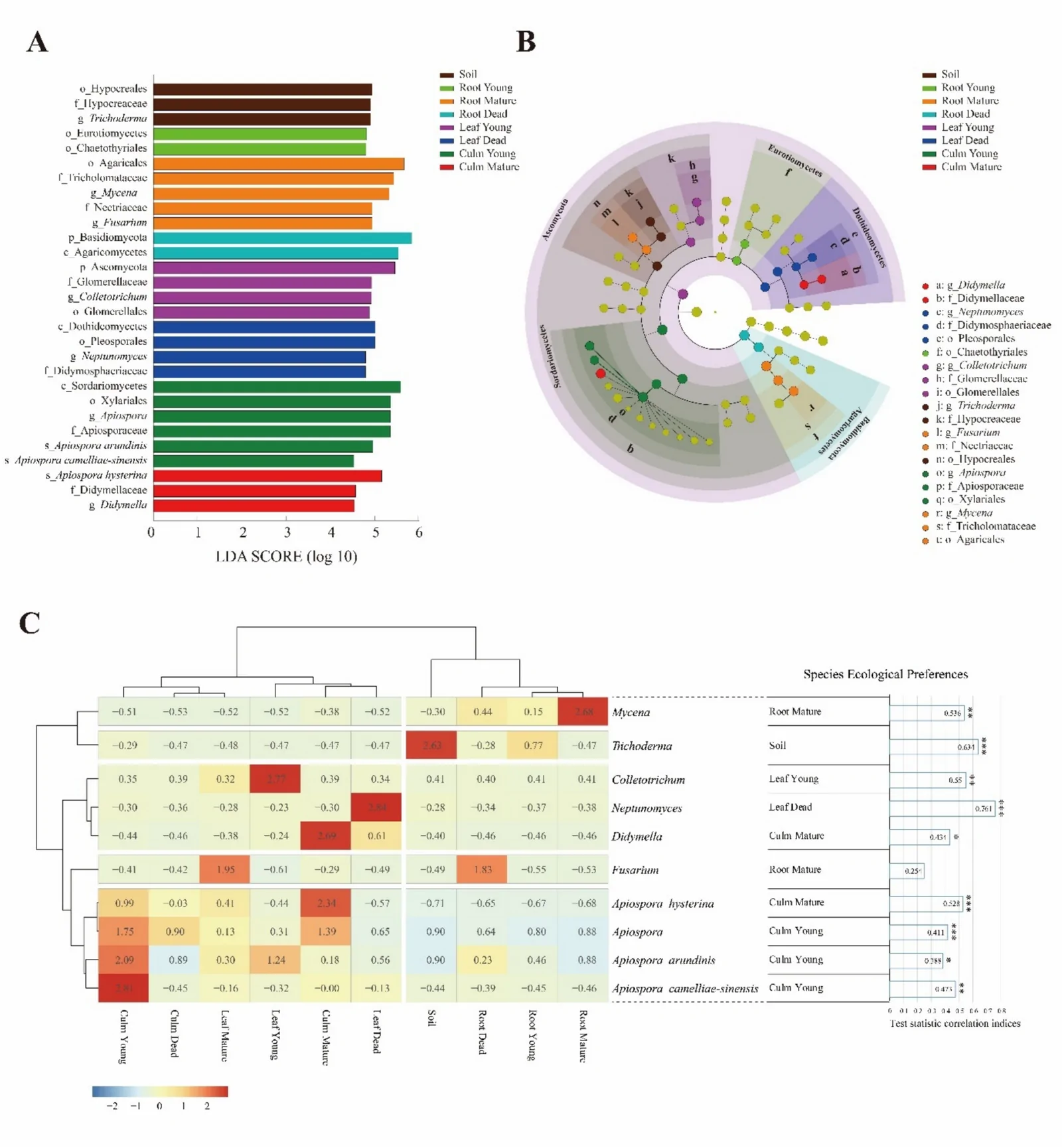
Ecological niches of bambusicolous endophytic fungi. Representative bambusicolous fungal taxa are shown in the linear discriminant analysis effect size (LEfSe) analysis between the stages of bamboo tissue and soil (LDA score > 2.0, *p* < 0.05). **A** Histogram represents the significantly representative taxa based on LDA effect sizes at taxonomic levels from phylum to species. **B** The cladogram shows the phylogenetic biomarkers of fungal lineages at different bamboo tissue stages and in soils. Significant biomarkers are highlighted and marked.** C** Heatmap clustering between relative abundances of representative bambusicolous taxa and stages of bamboo tissue and soil based on Spearman correlation. Ten representative taxa were selected for the LDA. The matrix values were normalized by row-wise Z-score normalization. The horizontal row shows the stages of bamboo tissue and soil growth, the vertical row represents the abundance of the bambusicolous fungal community, and the legend indicates the Z-score. The ecological preferences of the representative fungal species were calculated and presented as statistical correlation indices with *p* values. Significance levels are indicated by asterisks (*p < 0.05; **p < 0.01; ***p < 0.001; ns: not significant)

Representative fungal taxa were identified via LEfSe (Fig. 3A, B). Major genera such as *Apiospora* (young culms), *Didymella* (mature culms), *Colletotrichum* (young leaves), *Neptunomyces* (dead leaves), *Fusarium* and *Mycena* (mature roots), and *Trichoderma* (soil) exhibited significant associations (LDA > 2.0, *p* < 0.05). *Apiospora* species (*A. arundinis*, *A. camelliae-sinensis*, young culms; *A. hysterina*, mature culms) also showed significant preferences for living culms. This pattern reinforces the conclusion that tissue vitality (living vs. dead) plays a stronger role than developmental stage in structuring *Apiospora* communities.

Heatmap clustering (Spearman correlation) supported the observed taxa associations (Fig. 3C). High positive correlations were noted for *Mycena*, *Trichoderma*, *Colletotrichum*, *Neptunomyces*, *Didymella*, *Apiospora*, *A. hysterina*, *A. arundinis*, and *A. camelliae-sinensis*, supporting LEfSe findings. Major *Apiospora* species were significantly associated with living bamboo culms.

#### Trophic Mode Composition of Fungal Community

FUNGuild-based trophic mode assignments revealed ecological patterns within the endophytic fungal communities across bamboo compartments and stages. Among them, saprotrophs were the most abundant group, comprising 29.7% of the taxa (Additional File 2: Table S11). This was followed by pathotrophs at 22.9% and symbiotrophs at 12.7%, while 34.7% of the taxa remained unassigned. When examined at the compartment level, culms contained high proportions of saprotrophs (36.0%), pathotrophs (34.6%), and symbiotrophs (18.6%). Leaves hosted saprotrophic fungi (33.3%) and pathotrophic fungi (25.8%), with low symbiotrophs taxa (7.9%). In contrast, roots were dominated by unassigned fungi (57.6%), while soils primarily featured unassigned fungi (40.5%) and saprotrophic fungi (30.9%). Across different developmental stages, unassigned fungi were most prevalent in young bamboo (38.2%), while pathotrophs were more abundant in mature tissues (27.3%). Saprotrophs dominated in dead tissues (35.0%). Symbiotrophs remained relatively stable throughout the stages, ranging from 10.7% to 13.5%.

### Biological Activities

The biological activity analysis was conducted examining three bambusicolous *Apiospora* species using mycobiome analysis. *A. arundinis* (KUC21601 and KUC21792), *A. camelliae-sinensis* KUC21546, and *A. hysterina* (KUC21437 and KUC21435) exhibited high antioxidant activity in both the ABTS and DPPH assays (Additional File 2: Table S12). In the antifungal activity assay, *A. camelliae-sinensis* KUC21538 and *A. hysterina* (KUC21437 and KUC21435) inhibited the growth of *Botrytis cinerea* KUC21265. The extracts of *A. hysterina* (KUC21437 and KUC21435) inhibited the growth of *Colletotrichum gloeosporioides* KUC21266. However, the *A. arundinis* strains did not exhibit antifungal activity against phytopathogenic fungi.

The plant hormone ABA was detected in the fungal extracts of *A. hysterina* (KUC21437 and KUC21435) and *A. camelliae-sinensis* (KUC21536 and KUC21538) using MS/MS spectral matching with authentic standard compounds (Additional File 1: Fig. S5). GA derivatives (nodes 7451 and 7445) clustered with the node of GA (78,103) were detected in the extracts of the strains of *A. hysterina* (KUC21435, KUC21437, and KUC21438) (Additional File 1: Fig. S6). However, the LC–MS analysis of the standard and extract did not detect IAA.

### Genomic Analysis

#### Genome Sequencing, Assembly, and Annotation

Genomic analysis was conducted on the most dominant bambusicolous endophyte *Apiospora* species in this study, *A. hysterina* (Fig. 1A). The strain *A. hysterina* KUC21437 was selected due to its various biological activities and plant hormone production ability (ABA and GA). We obtained a 48.0 Mbp genome (GC content: 48.6%) with eight contigs (max. length and N50: 5.7 Mbp), excluding the mitochondrial genome. The genome assembly completeness in terms of gene content ranged from 97.7% (*Ascomycota*) to 99.2% (*Fungi*) with BUSCO 5.4.6 [35] and OrthoDB 10 [36]. A total of 14,433 protein-coding genes were identified via structural genome annotation.

### Trophic Lifestyle Prediction

#### CAZyme and CATAStrophy Analyses

**Fig. 4:**
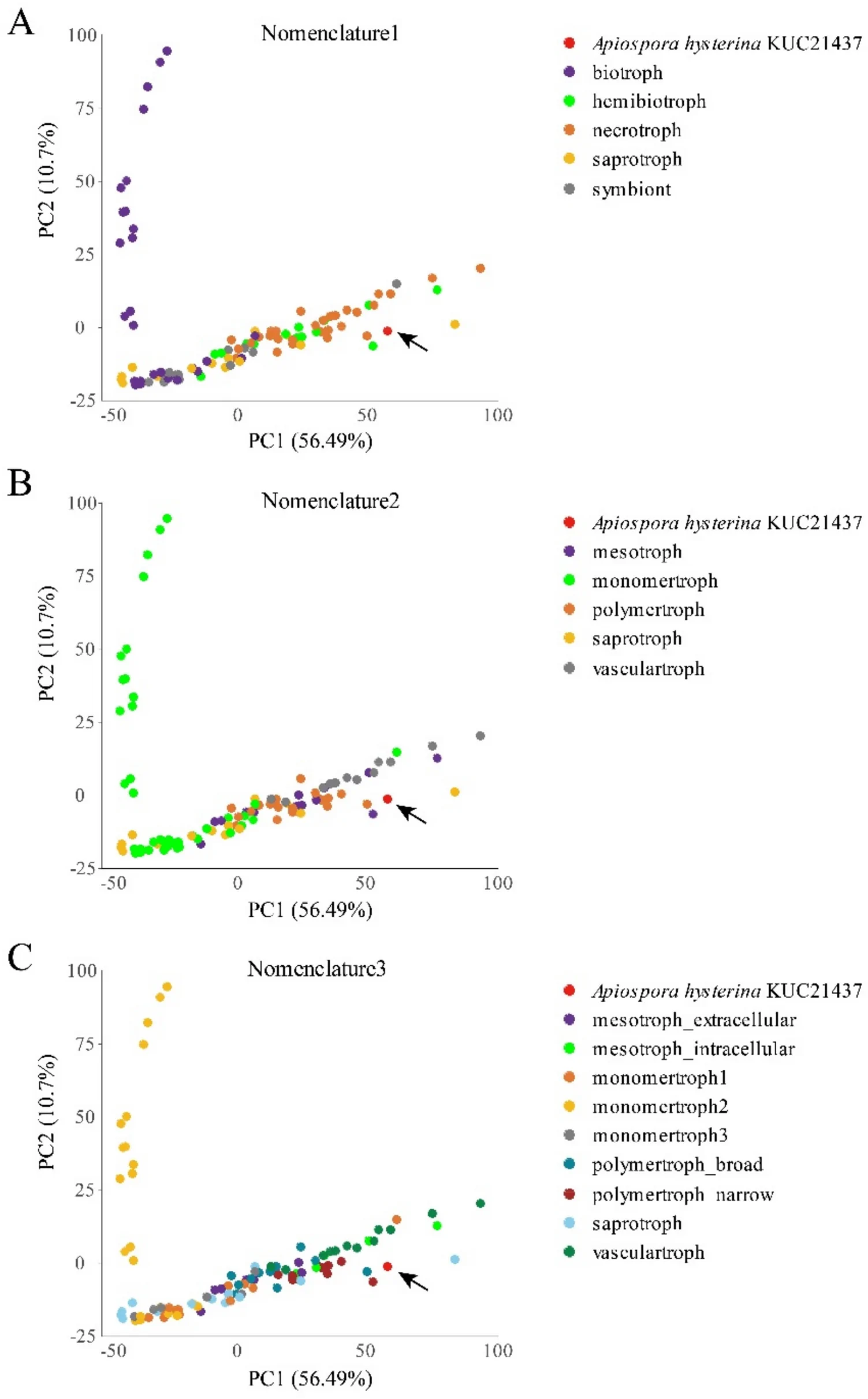
Carbohydrate-active enzyme (CAZyme) gene contents were compared across species to predict trophic lifestyle using principal component analysis (PCA) plots. PC1: principal component 1, PC2: principal component 2. The plot illustrates the CAZyme-inferred trophic phenotypes of 108 reference fungal and oomycete species assigned to different lifestyles: 29 biotroph, 14 hemibiotroph, 33 necrotroph, 14 saprotroph, and for symbiont based on the Nomenclature 1. The red arrows indicate *Apiospora hysterina* KUC21437. Three plots are indicated with **A** common trophic terms (Nomenclature 1), **B** five major trophic classes (Nomenclature 2), and **C** nine sub-classes (Nomenclature 3). The novel trophic classes were proposed based on Hane et al. [37]

The CAZyme analysis of the KUC21437 genome revealed 692 genes across 133 CAZyme families. Glycoside hydrolases (GH; 302 genes) dominated, with GH18 (chitinase; 21 genes) and AA7 (oxidase; 71 genes) being prevalent (Additional File 1: Fig. S7). CATAStrophy analysis predicted the lifestyle of *A. hysterina* KUC21437 as a “necrotroph,” representing the major trophic class under common trophic terms (Nomenclature 1) (Table 1). Principal component analysis revealed that the fungus was located close to the necrotrophs and hemibiotrophs and was significantly distinguished from the symbionts and biotrophs (Fig. 4A). According to the trophic mode classification (Nomenclature 2) of Hane et al. [37], the fungus was predicted to be a “vasculartroph (major trophic class)” based on the RCD score (Fig. 4B; Table 1). Moreover, it can be classified as “mesotroph_intracellular (major trophic class)” in novel trophic sub-classes (Nomenclature 3) based on the RCD score (Fig. 4C; Table 1). “Vasculotrophs” refers to fungal species commonly associated with diseases such as wilts, rots, or anthracnoses. “Mesotroph_intracellular” describes typical hemibiotrophic fungi that produce specialized structures, such as appressoria, to facilitate intracellular colonization.

**Table 1:** Summary of CATAStrophy classifications of Apiospora hysterina KUC21437 with relative centroid distance (RCD) scores ranging from 0 to 1

***Apiospora hysterina*KUC21437**	**Nomenclature 1**				
	**Necrotroph**	**Hemibiotroph**	**Saprotroph**	**Symbiont**	**Biotroph**
	**1**	0.90	0.39	0.26	0.0
	**Nomenclature 2**				
	**Vasculartroph**	**Mesotroph**	**Polymertroph**	**Saprotroph**	**Monomertroph**
	**1**	0.89	0.88	0.31	0.0
	**Nomenclature 3**				
	**Mesotroph_intracellular**	**Polymertroph_narrow**	**Vasculartroph**	**Polymertroph_broad**	**Mesotroph_extracellular**
	**1**	0.91	0.89	0.67	0.55

RCD score of 1 (bold and underlined) indicates memberships in a major trophic class, and score ≥0.95 (bold) predicts affinity for one or more trophic sub-classes

#### Effector Protein, Secretion System, and Virulence Factor Analysis

Predector analysis indicated that 8.6% of proteins of KUC21437 had effector scores > 0, exceeding the pathogen benchmark average (6.9%) and far above saprotrophs (3.5%) from previous research [34] (Additional File 2: Table S13). A total of 2335 proteins (16.2%) were predicted to be secreted, while 2883 proteins were predicted to be signal peptides (20.0%). Among the total proteome, 20.4% had matches to PHI-base, of which 9.1% had effector matches while 3.3% had lethal matches. Among the top 50 Predector candidates, 14 proteins showed homology to known effectors, higher than the pathogen average (6.2) from the previous research [34]. These included homologues of BgtAvrPm2 and BghBEC1011 (barley powdery mildew effectors), MoCDIP1 and MoCDIP4 (rice blast effectors), CfEcp6 and CfEcp2 (tomato leaf mold effectors), and VdAve1 (*Verticillium* wilt effector).

#### SM BGCs of *Apiospora hysterina*

A total of 81 secondary metabolite biosynthetic gene clusters (SM BGCs) were identified, including 25 type 1 polyketide synthases (T1PKS), 1 type 3 polyketide synthase (T3PKS), 18 terpenes, 12 non-ribosomal peptide synthetases (NRPS), 11 NRPS-like, and 14 hybrid genes (Additional File 1: Fig. S8). Among these, 25 BGCs showed sequence similarity to 22 known clusters in the MIBiG database, with 7 clusters displaying putative homology to 5 characterized SM BGCs based on antiSMASH similarity scoring (Additional File 2: Table S14). Notably, identified clusters included ACR toxin I, dimethyl coprogen, (R)-mellein, ACT toxin II, and AbT1.

#### Plant Hormone Synthesis Pathways

**Fig. 5:**
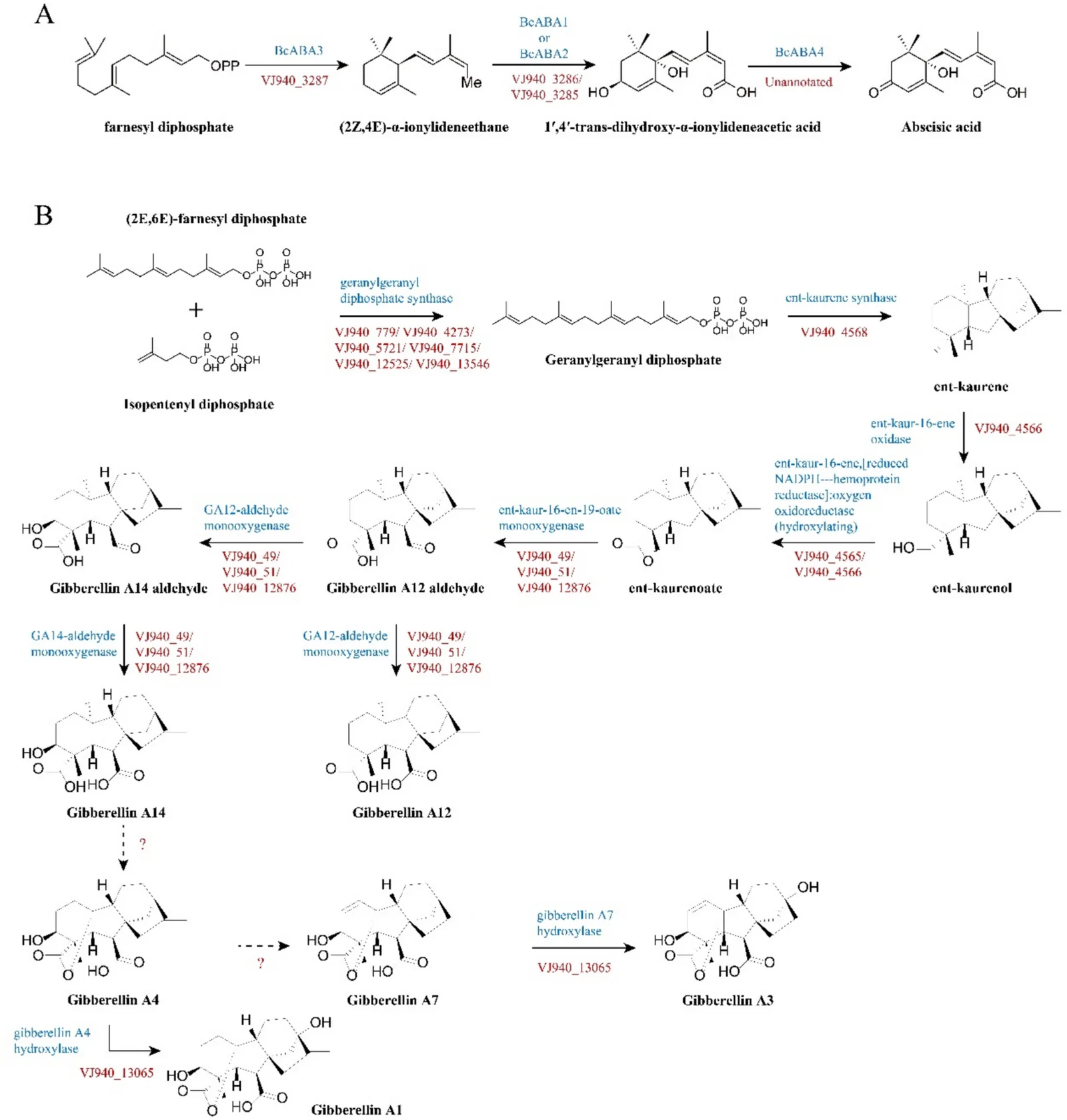
Putative plant hormone biosynthetic pathways predicted from genome analysis. **A** ABA and **B** GA in *A. hysterina* KUC21437 based on genome analysis. The predicted compounds in the pathway are linked by arrows, and the genome (red italics) and enzymes (blue) are denoted next to the arrow. The arrow with the dotted line indicates an unannotated pathway

Genome analysis predicted putative biosynthetic pathways for ABA and GA based on sequence homology to known enzymes in characterized biosynthetic routes (Fig. 5). The ABA biosynthesis gene cluster, homologous to *Botrytis cinerea*, was identified on chromosome 2 (1,571,658–1,592,948), containing homologues of *bcABA1*, *bcABA2*, and *bcABA3* (VJ940_3286, VJ940_3285, VJ940_3287), and *bcABA4* was located in an unannotated region (1,592,151–1,592,948) (Fig. 5A, B). Genes involved in GA biosynthesis from farnesyl diphosphate to GA14 were also detected. A gene cluster encoding enzymes from geranylgeranyl diphosphate to ent-kaurenoate was identified on chromosome 2 (6,063,250–6,076,058). Additional genes for GA derivative conversions (GA4 to GA1 and GA7 to GA3) were detected outside this cluster (VJ940_13065) (Fig. 5B).

## Discussion

Using mycobiome analysis, this study investigated the diversity of bambusicolous endophytic fungi across bamboo tissues and stages, with a focus on the genus *Apiospora*. The mycobiome analysis revealed that *Apiospora* is a dominant endophyte in aboveground parts of bamboo, particularly culms and leaves, from the young developmental stage to the dead state. Within the genus, *A. arundinis*, *A. camelliae-sinensis*, *A. hysterina*, and *A. rasikravindrae* emerged as core components of the bambusicolous endophytic community. Their persistence largely accounted for the observed nestedness, with *A. hysterina* showing an obligatory association with bamboo. In contrast, species such as *A. minutispora* and ITS_Apio_29, which were detected exclusively in dead bamboo tissues, appear to represent saprotrophic colonizers derived from outer tissues. Likewise, *Arthrinium phaeospermum*, found in both mature culm and dead root tissues, likely acts as both a pathotrophic and saprotrophic colonizer, consistent with its previous identification as a bamboo pathogen [38]. Moreover, *A. pseudohyphopodii* proliferated extensively in dead culms, suggesting potential saprophytic traits when bamboo tissues become senescent or die. These colonization patterns likely contributed to the raised turnover observed in dead tissues, reflecting species replacement.

Trophic mode assignments using FUNGuild further clarified the functional composition of bambusicolous endophytic fungi across bamboo compartments and developmental stages. Overall, saprotrophs were the largest guild (29.7%), but culms harbored a relatively high proportion of both pathotrophs (34.6%) and symbiotrophs (18.6%). Stage-specific analysis showed an increase in pathotrophs in mature tissues (27.3%), suggesting a possible shift toward latent pathogenic interactions, whereas symbiotrophs were maintained at a stable proportion across developmental stages (10.7–12.9%), pointing to a persistent but secondary mutualistic function. Taken together, these findings suggest that *Apiospora* species are potentially associated with bamboo culms and may adopt flexible lifestyles depending on host developmental stage.

To further explore the ecological roles, we employed a multidisciplinary approach that integrated mycobiome, biological activity tests (antioxidant, antifungal, and plant hormone production), and genomic analyses (CAZyme, effector/virulence factors, and SM BGCs). This framework enabled us to investigate not only the distribution of *Apiospora* but also its potential ecological roles in bamboo.

Based on the investigation, bambusicolous endophytic fungi, particularly *Apiospora*, can be interpreted within a framework of trophic plasticity, in which fungi may play multifaceted roles as mutualists or pathogens. Although our data cannot fully verify this model, the concept of “balanced antagonism” offers a valuable perspective to contextualize the dual and ambiguous roles of endophytes in bamboo ecosystems.

In this study, we primarily focused on *A. hysterina* to investigate its potential ecological roles in bamboo. Through mycobiome analysis, it was identified as the dominant endophytic fungi living in young culms and leaves but was absent from the soil. This distribution pattern indicates a strong ecological specificity to the host plant of this fungus compared to other fungi. Furthermore, its ability to produce two plant hormones (ABA and GA) and exhibit antioxidant and antifungal activities highlights its potential as a key player in the bamboo ecosystem, serving as a mutualist. Furthermore, genomic analyses revealed a substantial repertoire of effector proteins and virulence factor homologues, indicating latent pathogenic potential. These findings, together with its mutualistic traits, suggest that *A. hysterina* may undergo trophic lifestyle shifts in response to host condition and tissue vitality, aligning with the concept of fungal trophic plasticity.

### Putative Pathogenic Characteristics of *A. hysterina*

Some *Apiospora* species have been historically identified as plant pathogens that cause diseases such as culm blight (*A. kogelbergensis*) [32], culm base rot (*Arthrinium phaeospermum*) [38], culm staining (*Apiospora indica*) [38], and dieback (*Apiospora* sp.) [38] in bamboo. In addition to bamboo, *Apiospora* species have also been reported as plant pathogens in other crops, such as reddish-brown discoloration in sugarcane flesh [39].

Our genomic analyses of *A. hysterina* support its pathogenic potential, consistent with previous studies. Specifically, the CAZyme content of *A. hysterina* KUC21437 suggests a trophic lifestyle consistent with a “necrotroph” (Nomenclature 1), “vasculartroph” (Nomenclature 2), and “mesotroph_intracellular” (Nomenclature 3) with high RCD scores (RCD score > 0.95) (Table 1). These classifications imply that the CAZyme content of *A. hysterina* is well suited for plant infections, including vascular invasion and intracellular penetration via appressorial structure formation [37]. However, appressorial structures have been observed in phylogenetically close species, such as *A. pseudohyphopodii* and *A. yunnana* [30]. Direct evidence for such structures in *A. hysterina* has not yet been obtained. Nonetheless, its phylogenetic proximity suggests the potential for similar capabilities, requiring further research.

Predector analysis indicated the pathogenic potential of *A. hysterina* KUC21437. Specifically, 8.6% of proteins received effector scores above zero, and 16.2% were predicted to be secreted. Moreover, more than 20% of the proteome showed homology to PHI-base entries, including 14 proteins similar to experimentally validated effectors such as AvrPm2 from *Blumeria graminis* f. sp. *tritici* and BEC1011 from *B. graminis* f. sp. *hordei*, both linked to powdery mildew pathogenesis [40, 41]; MoCDIP1 and MoCDIP4 from *Magnaporthe oryzae*, associated with cell death induction and suppression of rice immunity [42]; Ecp6 and Ecp2 from *Cladosporium fulvum*, which interfere with chitin-triggered immunity and contribute to virulence [43]; and VdAve1 from *Verticillium dahliae*, reported to play a role in strain-specific interactions Ve1 [44]. The presence of secreted proteins with effector-like scores suggests that *A. hysterina* KUC21437 may utilize a dual strategy of pathogenesis, combining enzymatic degradation of host cell walls (via CAZymes) with molecular interference in host immunity (via effectors).

BGC analysis using antiSMASH identified putative secondary metabolite gene clusters with sequence similarity to characterized pathogenicity-associated clusters in *A. hysterina* KUC21437 (Additional File 2: Table S14). These included clusters resembling previously identified plant pathogenicity-related BGCs in *Apiospora* species, including ACR toxin I, dimethyl coprogen, and (R)-mellein BGCs. In addition, a cluster showing similarity to the ACT toxin II BGC, an essential host-selective toxin responsible for the pathogenesis of *Alternaria alternata* [45], was detected for the first time in *A. hysterina* KUC21437. ACT toxin II is known to cause *Alternaria* brown spots in tangerines (*Citrus reticulata* Blanco), grapefruits (*Ci. paradisi* Macfad.), and their hybrids [46]. However, the actual product of these BGCs should be further validated with metabolite analysis, as antiSMASH similarity reflects BGC gene content homogeneity rather than confirmed metabolite production.

While *A. hysterina* has been reported as the causal agent of severe leaf spots in faba bean (*Vicia faba*), leading to symptoms that eventually result in leaf abscission [47], no evidence to date links disease symptoms in bamboo. Therefore, further research on secondary metabolites and pathogenicity bioassays is necessary to fully understand the role of bamboo pathogens.

### Mutualistic Role of *A. hysterina*

Plant–fungal interactions must consider not only genetic traits but also biological activities. Thus, investigating the biological activities associated with symbiotic interactions is a fundamental approach for elucidating the critical role of endophytic fungi in the health and resilience of their hosts [48]. Through biological activity analyses, we identified mutualistic traits in *A. hysterina* that may benefit bamboo in addition to its pathogenic potential.

The antioxidant production capability of *Apiospora* species, as demonstrated in this study, highlights their ability to reduce reactive oxygen species (ROS). This reduction can enhance plant stress tolerance under conditions such as drought [49], metals [50], pathogens [51], and salinity [49]. Moreover, antioxidants play a critical role in stress signaling [52], facilitating chemical communication between the host and an asymptomatic endophyte, including an avirulent pathogen. This enables the host to rapidly respond to pathogenesis and differentiate between mutualistic and pathogenic interactions [53].

The antifungal activity of *A. hysterina* KUC21437, observed under controlled conditions against plant pathogens, may hint at a potential role as a mutualistic endophyte. BGC analysis revealed similarity to antibiotic-related BGCs, including AbT1, a precursor for synthesizing Aureobasidin A (AbA) in *Aureobasidium pullulans* [54]. AbA is a cyclic depsipeptide antibiotic with potent antifungal activity against various plant fungal pathogens, including *Aspergillus fumigatus*, *Aspergillus nidulans*, *Aspergillus niger*, *Aspergillus oryzae*, *Blastomyces dermatitidis*, *Candida albicans*, *Cryptococcus neoformans*, *Histoplasma capsulatum*, and *Schizosaccharomyces pombe* [54–56]. Although further studies are required to confirm whether this activity occurs in natural hosts, these findings indicate that *Apiospora* may contribute to the mycobiome’s protective functions against external threats, particularly under stress conditions such as pathogen invasion or abiotic challenges, where antioxidant and antifungal metabolites could enhance host resilience.

Mycobiome analysis revealed that *A. hysterina* exhibits a strong ecological association with bamboo as an endophyte, suggesting it may form an obligatory association with its host, showing higher host specificity than other fungal taxa. Its pronounced increase in abundance in mature bamboo could indicate a latent pathogenic phase, although no visible disease symptoms were observed. The early and persistent presence of *A. hysterina* in young bamboo tissues, coupled with its potential ability to produce diverse bioactive compounds, supports the hypothesis that it may also act as an endophytic symbiont engaged in a mutualistic relationship with the host.

### Multifaceted Roles and Trophic Mode Switching

The dual roles of *Apiospora* as mutualists and pathogens suggest a complex ecological strategy. In this study, *A. hysterina* was unexpectedly dominant in bamboo tissues at all stages, including the early young stages, without visible black spots. This observation, combined with the absence of *Apiospora* in the surrounding soil, suggests that *Apiospora* establishes itself as an endophyte before pathogenic symptoms manifest.

The detection of plant hormones ABA and GA in *Apiospora* extracts, combined with computational identification of putative biosynthetic genes, suggests hormone production capabilities that may contribute to its multifaceted ecological roles. In this study, GA derivatives and ABA were detected in *A. hysterina* under controlled conditions, but IAA was not detected. While IAA is strongly associated with symbiosis in host plants, both GA and ABA have roles linked to pathogenicity and mutualism [57, 58]. GA regulates various plant processes, such as seed development [59], pollen tube growth [59], plant development [60], and internode elongation. Fungal GA production has been reported to enhance seed germination under salt stress and may facilitate plant carbon sink activity in infected cells [61].

We report that *Apiospora* species produce ABA. Fungal ABA has been identified as a virulence factor that suppresses plant immune responses [62, 63] and is associated with the establishment of symbiosis with the host plant [64]. Although the specific role of ABA in *Apiospora* remains unclear, it may help infect bamboo species by reducing plant resistance.

Collectively, we propose that *Apiospora* initially functions as a mutualistic endophyte that promotes bamboo growth and stress tolerance. However, it may transition to a pathogenic state under specific environmental or host conditions. Such trophic mode switching has been well documented in other fungal endophytes, such as dark septate endophytes, whose interactions shift from mutualism to parasitism based on nutrient availability or host performance [65]. This dynamic highlights that fungal–host interactions are not fixed but are shaped by biochemical communication and environmental cues, consistent with the balanced antagonism framework [66].

Similar examples in bamboo-endophyte systems underscore this multifaceted role. For instance, *Shiraia* sp. isolated from bamboo seeds (*Phyllostachys edulis*) act as antimicrobial endophytes but are also significant bamboo pathogens [67]. Similarly, *Aciculosporium take*, an endophytic bambusicolous fungus, promotes shoot growth via auxin production but causes witch’s broom disease [68]. These cases show that bambusicolous fungi such as *Shiraia* and *Aciculosporium* exhibit both mutualistic and pathogenic behaviors. While the underlying mechanisms remain unknown, their ecological plasticity mirrors the multifaceted lifestyle we propose for *Apiospora*.

## Conclusion

This study highlights the multifaceted roles of *Apiospora hysterina* in bamboo ecosystems as endophytes, demonstrating their potential as mutualists and pathogens. The coexistence of these roles underscores the complexity of plant–fungal interactions, emphasizing the necessity of exploring fungal behavior across different ecological contexts. Understanding these dynamics could have broader implications for managing bamboo health and leveraging endophytes for sustainable bamboo cultivation. Furthermore, this research highlights the significance of adopting a holistic approach (mycobiome, chemical, and genomic analysis) in studying plant–fungal relationships involving bamboo *Apiospora* and the need for further research in this field.

## Supplementary Information

Below is the link to the electronic supplementary material.

ESM 1 (DOCX 8.12 MB)

ESM 2 (XLSX 93.9 KB)

ESM 3 (DOCX 42.2 KB)

## Data Availability

The whole genome sequence of **A. hysterina** KUC21437 generated and analyzed during the current study are available in the NCBI repository, NCBI BioProject PRJNA1060443 with accession number JAYMYE000000000. The amplicon sequencing data are available in the NCBI SRA repository, BioProject PRJNA1065571.
