# Supplementary material for: Exploring Multifaceted Roles of Bambusicolous Apiospora in Phyllostachys bambusoides
Source: Microb Ecol. 2025 Nov 5;88:115. doi: 10.1007/s00248-025-02631-z (PMC12586400; doi:10.1007/s00248-025-02631-z)
Supplement: Supplementary file 2 [file 248_2025_2631_MOESM3_ESM.docx]

**Methods**

**Bambusicolous fungal microbiome**

**Sample treatment**

Sample surface sterilization was conducted following a modified protocol described previously [1]. Samples were washed with tap water and submerged in 70% (v/v) ethanol (Duksan Chemical Co., Ltd., Ansan, Korea) for 3 min. Subsequently, the samples were immersed in 2.5% (v/v) sodium hypochlorite solution (Samchun Pure Chemical Co., Ltd., Pyeongtaek, Korea) for 5 min before rinsing the surface with 70% ethanol for 30 s and washing thoroughly with sterile distilled water. The absence of colonies in the final rinse water culture confirmed the successful removal of epiphytes. To verify that surface sterilization was effective in eliminating external infectant, sterilized bamboo tissue’s surfaces were imprinted onto culture media, and the absence of fungal growth was confirmed. After surface sterilization, the samples were crushed and powdered in liquid nitrogen using a Freeze Mill (SPEX 6875D) at NICEM (National Instrumentation Center for Environmental Management), Seoul National University. The vials and steel impactors of the frozen mill were rinsed with 70% ethanol before proceeding with the machine to avoid sample contamination during the crushing process.

**Amplicon library preparation and sequencing**

Genomic DNA was extracted from each powdered bamboo tissue and soil using a DNeasy Powerlyzer PowerSoil Kit (Qiagen, Hilden, Germany) according to the manufacturer’s instructions. PCR was conducted to amplify the extracted DNA with KAPA HiFi HotStart ReadyMix (KAPA Biosystems, Wilmington, MA, USA) and the primer set ITS3 (5′- GCATCGATGAAGAACGCAGC-3′) and ITS4 (5′-TCCTCCGCTTATTGATATGC-3′) [2, 3]. The purification of PCR products was conducted using the Agencourt AMPure XP system (Beckman Coulter Genomics, Brea, CA, USA). Amplicon libraries were constructed using an Illumina MiSeq platform with rDNA *ITS2* gene amplicons. Sequencing was performed by LAS Inc. (Gimpo, Korea) using the Illumina MiSeq system with 2×300-bp, paired-end reads.

**Read processing and identification**

The rDNA ITS2 amplicon sequence data sets were processed using QIIME2 (version 2021.8.0) [4]. The raw sequence data were demultiplexed using the QIIME2 demux plugin. Quality filtering and denoising were performed with DADA2 via the QIIME2 dada2 plugin, removing low-quality (QV < 20), short (< 200 bp), and chimeric sequences [5]. Molecular operational taxonomic units (mOTUs) were clustered with a 99% threshold identity using the QIIME2 VSEARCH cluster-features-open-reference plugin [6]. Low coverage mOTUs consisting of < 10 sequences were discarded.

The taxonomy of mOTUs was initially assigned using the modified UNITE version 8.3 database (UNITE-Resources. https://unite.ut.ee/repository.php. Accessed on July 20, 2022). The ITS sequences related to *Apiospora* species, including *Arthrinium* and *Nigrospora* species, in the UNITE database, were replaced and added to verified ITS sequences downloaded from the GenBank database (https://www.ncbi.nlm.nih.gov/genbank/) based on previous studies to enhance the precision of *Apiospora* species assignment.

Based on the taxonomic assignment, two taxonomic levels were selected to analyze the variation of the bambusicolous endophytic fungal community in the bamboo compartments and stages. The genus-level community (GLC) was selected to analyze the proportion of *Apiospora* in the bamboo. The *Apiospora* species-level community (ALC) was selected to analyze the species that act as endophytes in bamboo.

To identify *Apiospora* species, phylogenetic analysis was conducted with *Apiospora* mOTU and reference sequences of *Apiospora* and related genera from the GenBank database (Additional file 2: Table S1). mOTUs with short sequence lengths (< 260 bp) were excluded from the phylogenetic analysis. The *Apiospora* mOTU and reference sequences were aligned using MAFFT 7.130 [7]. The Maximum Likelihood tree was inferred using the RaxML version 8 with the GTR+G model and 1,000 bootstrap replicates on the CIPRES web portal [8].

**Bioinformatics**

The “vegan” package was applied to conduct α- and β-diversity analyses to evaluate the variation of bambusicolous fungal communities according to the bamboo compartments (bamboo culm, leaf, root, and bamboo forest soil) and stage (young, mature, and dead) [9]. The distribution of each dataset was tested using the Shapiro–Wilk test, Durbin–Watson test, and Bartlett test for homogeneity of variances. The one-way ANOVA and Tukey’s Honest Significant Distance (HSD) tests were adjusted with multiple comparisons for normally distributed data. Kruskal–Wallis and Dunn’s tests with Benjamini-Hochberg (BH) methods were adjusted for non-normally distributed data. The fungal community variation was visualized using principal coordinates analysis (PCoA) based on Bray-Curtis dissimilarities. The significant (*p* < 0.05) factors and vectors were fitted on the plots using the goodness-of-fit statistics (r^2^). The adjusted p-values were corrected using the Bonferroni correction method.

Prior to permutational multivariate analysis of variance (PERMANOVA), beta dispersion was evaluated using “betadisper()” function in the “vegan” package to test for homogeneity of multivariate dispersions. The most influential factor in explaining the variation of communities was examined by PERMANOVA and pairwise PERMANOVA based on the Bray-Curtis distance metrics with 999 permutations via the “adonis2()” function in the “vegan” package. To further interpret the sources of β-diversity, it was partitioned into turnover, nestedness, and total dissimilarity. Subsequently, distance-based redundancy analysis (dbRDA) using Bray-Curtis dissimilarities was applied to identify significant explanatory factors. The Venn diagram software in the Bioinformatics & Evolutionary Genomics platform (http://bioinformatics.psb.-ugent.be/webtools/Venn/) was used to reveal the overlapped bamboo endophytic fungi between the bamboo compartments and between each tissue stage. To distinguish significantly representative bambusicolous fungal taxa according to the bamboo tissue stages and soils, a linear discriminant analysis (LDA) combined with effect size (LefSe) was performed on the Huttenhower galaxy server (LefSe, http://huttenhower.sph.harv-ard.edu/galaxy) [10]. Heat map analysis was conducted using representative taxa determined by LDA. The heat map was constructed with the “pheatmap” package version 1.0.12 [11]. Heat map clustering between the relative abundances of bambusicolous endophytic fungi and bamboo tissue stages and soil was conducted based on Spearman correlation. The data matrix was Z-score-normalized by row. Indicator species analysis was conducted to determine the species ecological preference of the representative taxa via “multipatt(data, group, func=”r.g”, control= how(nperm=999))” in the “Indicspecies” package [12]. All statistical analyses were performed with R software version 4.2.0 [13].

**Trophic Mode Analysis**

Trophic mode assignments for fungal OTUs were performed using FUNGuild v1.1 [14] on 6,903 fungal OTUs across 89 samples from different plant compartments and stages. For fungi assigned to multiple trophic modes, we implemented weighted guild assignment by proportionally dividing OTU abundance among all assigned trophic modes (e.g., “Pathotroph–Saprotroph” split as 50% each). Trophic mode proportions were compared across compartments and stage.

**Bambusicolous *Apiospora* strains**

Bambusicolous *Apiospora* strains were obtained from the Korea University Culture Collection (KUC). These strains were isolated, identified, and deposited in a previous study [15], which reported 242 bambusicolous *Apiospora* strains from various bamboo materials collected in bamboo forests in Korea. In this study, we employed nine bambusicolous *Apiospora* strains, representing three *Apiospora* species isolated from dead bamboo culms and branches. Based on the results of mycobiome analysis, we determined that these three *Apiospora* species on dead bamboo (*Phyllostachys* spp.) materials could be derived from the inner tissue of bamboo. Consequently, these strains were used for further investigations of their biological activities or genomic analyses. The three *Apiospora* species were *A. arundinis* (KUC21601, KUC21792, and KUC21722), *A. camelliae-sinensis* (KUC21546, KUC21536, and KUC21538), and *A. hysterina* (KUC21437, KUC21435, and KUC21438). The strain information is detailed in Additional file 2: Table S2.

**Biological activity and metabolite analysis**

***Apiospora* extract preparation**

The fungal extract was prepared as previously described [16]. Bambusicolous *Apiospora* strains were cultured on potato dextrose agar at 25^◦^C for 7 days in the dark. The fungal biomass was extracted with methanol for 24 h, and the solution was filtered using Whatman® No. 1 filter paper (Cytiva, Marlborough, MA, USA). Subsequently, the filtrate was concentrated using a rotary vacuum evaporator at 37^◦^C. After concentration, the condensed residues were dissolved in a 1:1 mixture of ethyl acetate (EtOAc) (Samchun Pure Chemical Co., Ltd., Pyeongtaek, Korea) and distilled water. The EtOAc fraction of the solution was then obtained and concentrated again following the aforementioned method. The EtOAc extracts were stored at –20^◦^C.

**Antioxidant analysis with ABTS and DPPH radical scavenging ability**

A 7-mM ABTS (2.2′-azino-bis-3-ethylbenzothiazoline-6-sulfonic acid, Sigma-Aldrich, Inc., St. Louis, MO, USA) solution was oxidized with 2.45 mM potassium persulfate for 24 h in the dark at room temperature. Before the next step, the ABTS^•+^ solution was diluted with 1× phosphate-buffered saline (Biosesang, Yongin, Korea) to record an absorbance value of 0.70 (±0.02) at a wavelength of 734 nm. Then, 990 µL of ABTS solution and 10 µL of each fungal extract were mixed in a cuvette, and the absorbance was measured at 734 nm after 6 min in the dark. Trolox (6-hydroxy-2,5,7,8-tetramethylchroman-2-carboxylic acid, Sigma-Aldrich) was used as a positive control.

A 150-µM DPPH (2,2-diphenyl-1-picrylhydrazyl, Sigma-Aldrich) solution was dissolved in 80% methanol. Each fungal extract (22 µL) was mixed with DPPH solution (200 µL) in a 96-well plate. The mixture was stored at room temperature for 30 min in the dark, and the absorbance was measured at 520 nm. L-Ascorbic acid (Sigma-Aldrich) was used as a positive control.

**Antifungal activity test**

The disk diffusion method was used to screen the antifungal activities of bambusicolous *Apiospora* against three phytopathogens: *Botrytis cinerea* KUC21265, *Colletotrichum gloeosporioides* KUC21266, and *Fusarium oxysporum* KUC21267. The three phytopathogens were selected based on their broad host-range pathogenicity [17, 18]. Moreover, *C. gloeosporioides* and *F. oxysporum* are bamboo disease fungi that cause leaf spot or culm rot [19]. After cultivation, the phytopathogens were transferred to new Malt Extract Agar media (2% malt extract (Difco, MI, USA) and 1.5% Bacto agar (Difco), and the fungal extract (2.5 mg/mL concentration, 10 µL volume) was inoculated beside the phytopathogens. After 48 h of cultivation at room temperature, the growth inhibition of the pathogens was determined.

**Analysis of plant hormone production**

To evaluate IAA production, strains were cultured in Czapek broth media (0.05% potassium chloride (Yakuri Pure Chemicals, Kyoto, Japan), 1% dextrose (Difco), 0.05% magnesium sulfate (Shinyo Pure Chemicals Co., Ltd., Osaka, Japan), 1% Bacto Peptone (Difco), and 0.001% Ferrous sulfate heptahydrate (Showa Denko KK, Tokyo, Japan; pH 7.0 ± 0.2) with L-tryptophan (Sigma-Aldrich; 1 mg/mL) at 25°C for 7 days under darkness in a shaking incubator at 120 rpm. After cultivation, the culture supernatants were mixed with two volumes of EtOAc for extraction. The extracts were dissolved to 1 mg/mL in methanol and then stored at –20°C.

To evaluate the production of (S)-(+)-Abscisic Acid (ABA) and Gibberellic Acid A3 (GA), the prepared *Apiospora* extracts were used as described above. The fungal extracts and three standard compounds, including IAA (Sigma-Aldrich), ABA (Tokyo Chemical Industry Co., Tokyo, Japan), and GA (RPI, Chicago, IL, USA), were analyzed using liquid chromatography with tandem mass spectrometry (LC MS/MS) with the Thermo Scientific™ Vanquish™ HPLC system (Thermo Fisher Scientific, Waltham, MA, U.S.) coupled to an Orbitrap Exploris 120 mass spectrometer (Thermo Fisher Scientific), which was equipped with an electrospray ionization interface (ESI). A YMC Triart C18 (2.1 × 100 mm, 1.9 μm) was used. H2O (A) and MeCN (B) were eluted at a flow rate of 0.3 mL/min with a linear gradient of 20%–100% B (0–7 min). In addition, the ESI conditions were set as follows: capillary temperature of 275°C, capillary voltage of 35.0 V, spray voltage of 3.5 kV and sheath gas flow rate of 50.0 arb. The acquired data file was exported in .mzXML format by the application of MS-convert software, which is part of the ProteoWizard package. The molecular networks were generated using Global Natural Products Social Molecular Networking (http://gnps.ucsd.edu). The parameters were set as follows: product ion tolerance of 0.02 Da, precursor-ion mass tolerance of 0.2 Da, and fragment ions below six counts were removed from the MS/MS spectra. The molecular networks were generated using four minimum matched peaks and a cosine score of 0.65. The results were visualized using Cytoscape 3.7.2 software.

**Whole genome analysis of major bambusicolous *Apiospora***

***Apiospora* culture and genomic DNA extraction**

Genomic analysis was conducted with a major bambusicolous *Apiospora* strain selected based on microbiome and biological activity analyses to investigate the genomic features of this endophytic species. A bambusicolous *Apiospora* strain was grown in 500 mL of potato dextrose broth medium for 7 days at 25°C in the dark. DNA was extracted using the cetyltrimethylammonium bromide method with some modifications [19]. The quality of the DNA was assessed using an Agilent 2100 Bioanalyzer (Agilent Technologies, Palo Alto, CA, United States) with a DNA 1000 chip.

**Library construction and whole genome sequencing (WGS)**

A DNA library with approximately 20-kb fragment sizes was constructed, and WGS was performed using the PacBio Sequel and NovaSeq6000 platforms at Macrogen Co., Ltd. (Seoul, Korea). Meanwhile, a single-molecule real-time (SMRT) library was constructed and sequenced with a single SMRT cell. PacBio ccs 6.4.0 (https://github.com/PacificBiosciences/ccs) with the min-passes = 0 and min-rq = 0 options used to solve potential HiFi reads, whereas the HiFiAdapterFilt 2.0.1 was used to remove remnant adapter sequences [21]. *De novo* assembly was performed using Flye 2.9.2 [22]. For NovaSeq, Illumina sequencing was performed using a 150×2 bp paired-end system. Quality filtering and adapter trimming for Illumina reads were performed with fastp 0.23.2 [23]. Polishing with Illumina reads was performed with four rounds of Racon 1.5.0 and another four rounds of Hapo-G 1.3.1 [24, 25]. Assembled genomes were inspected with Tapestry 1.0.0, and contigs with low depth were considered contaminant contigs and were manually removed [26].

**Genome annotation**

The mitochondrial genome was removed manually. Repeats were modeled and masked for nuclear genomes using RepeatModeler v2.0.3 and RepeatMasker v4.1.2 [27, 28]. Ab initio annotations on masked genomes were performed using BRAKER2 with the odb10v1 database fungus option [29]. Homolog-based gene prediction was performed with GeMoMa 1.7.1, using model annotation of *Apiospora koreana* KUC21332 [30]. The structural annotations obtained by both methods were merged with the GAF function of GeMoMa with equal scoring. The merged annotations were finalized using the AnnotationFinalizer function of GeMoMa. tRNA genes were annotated using tRNAscan-SE 2.0.9, and rDNA regions were annotated using the Rfam database and infernal 1.1.4 [31, 32]. For CAZyme and gene cluster analysis, protein sequences were obtained by applying the agat_sp_keep_longest_isoform.pl function of AGAT 1.4.0 and gffread 0.12.7 [33, 34]. To validate the predicted protein-coding genes and identify potential functions, the easy-search workflow of MMseqs2 13.45111 with the “--greedy-best-hits 1” option was employed against the eggNOG 5.0.2 database with eggNOG-mapper 2.1.8 [35–37].

**Trophic lifestyle classification according to CAZyme profiles**

The run_dbcan 3.0.6 function of dbCAN3 with the HMMER search option was employed to predict and annotate the presence of CAZyme-related genes [38, 39]. The trophic lifestyle was predicted using the CAZyme Assisted Training And Sorting of trophy (CATAStrophy) 0.1.0 prediction tool [40]. The CATAStrophy pipeline (https://github.com/ccdmb/catastrophy-pipeline) was employed with the options -profile conda and –dbcan_version 10. The trophic lifestyle nomenclature in CATAStrophy (including biotrophs, hemibiotrophs, necrotrophs, saprotrophs, and symbionts) was used to define the trophic lifestyles of *Apiospora* species. The 108 reference fungal taxa contained in the default database (HMMdb-v10, model_v10_20211001.npz) and their corresponding lifestyle assignments under Nomenclature 1–3 are summarized in Additional file 2: Table S15. Principal components PC1 and PC2, which separated most training set species according to lifestyle, were selected for visualization.

**Effector protein and virulence factor analysis.**

Putative effector proteins were predicted from the complete proteome of *Apiospora hysterina* KUC21437 using Predector 1.2.7 [41]. Predector was performed with default option, except for SginalP 6.0g changed to SignalP 6.0h due to unavailability. The results were compared with data from previous research [41].

**Analysis of secondary metabolite biosynthetic gene clusters (SM BGCs)**

The SM BGCs were predicted using antiSMASH 6.1.1 locally, with the taxon fungi option and all possible search options appended [42]. The analysis was performed on repeat-masked genomic sequences with gene annotations in GFF format, using the following parameters: --taxon fungi --cassis --clusterhmmer --tigrfam --asf --cc-mibig --cb-general --cb-knownclusters --cb-subclusters --pfam2go --rre --smcog-trees.

**Reconstruction of the plant hormone synthesis pathway**

Genome-scale model (GEM) of *A. hysterina* KUC21437 was constructed using RAVEN Toolbox 2.8.4 [43]. The reference GEM of the closest available species, *Eutypa lata* and the MetaCyc database were applied to construct the GEM [44]. To annotate missing genes for ABA synthesis, genes comprising the ABA synthesis gene cluster of *B. cinerea* SAS56, *bcABA1, bcABA2, bcABA3*, and *bcABA4* (XM_001553921.1, XM_001553920.1, XM_001553924.1, XM_001553919.2) were downloaded from GenBank, and BLASTx [45] analysis was performed against the KUC21437 genome with Geneious 9.1.8 [46]. The final ABA and GA synthesis pathways were constructed by manually combining the GEM, BLAST, and reference search results and were visualized using ChemDraw software.

**List of references**

1.	Barra PJ, Inostroza NG, Acuña JJ, Mora ML, Crowley DE, Jorquera MA (2016) Formulation of bacterial consortia from avocado (*Persea americana* Mill.) and their effect on growth, biomass and superoxide dismutase activity of wheat seedlings under salt stress. Appl Soil Ecol 102:80–91. <https://doi.org/10.1016/j.apsoil.2016.02.014>

2.	White TJ, Bruns T, Lee S, Taylor J (1990) Amplification and direct sequencing of fungal ribosomal RNA genes for phylogenetics. In: Innis MA, Gelfand DH, Sninsky JJ, White TJ (eds), PCR protocols: A guide to methods and applications. Elsevier, New York, pp 315–322. [https://doi.org/10.1016/B978-0-12-372180-8.50042-1](https://doi.org/10.1016/b978-0-12-372180-8.50042-1)

3.	Bellemain E, Carlsen T, Brochmann C, Coissac E, Taberlet P, Kauserud H (2010) ITS as an environmental DNA barcode for fungi: an in silico approach reveals potential PCR biases. BMC Microbiol 10:189. <https://doi.org/10.1186/1471-2180-10-189>

4.	Bolyen E, Rideout JR, Dillon MR, Bokulich NA, Abnet CC, Al-Ghalith GA, Alexander H, Alm EJ, Arumugam M, Asnicar F, Bai Y, Bisanz JE, Bittinger K, Brejnrod A, Brislawn CJ, Brown CT, Callahan BJ, Caraballo-Rodríguez AM, Chase J, Cope EK, Da Silva RD, Diener C, Dorrestein PC, Douglas GM, Durall DM, Duvallet C, Edwardson CF, Ernst M, Estaki M, Fouquier J, Gauglitz JM, Gibbons SM, Gibson DL, Gonzalez A, Gorlick K, Guo J, Hillmann B, Holmes S, Holste H, Huttenhower C, Huttley GA, Janssen S, Jarmusch AK, Jiang L, Kaehler BD, Kang KB, Keefe CR, Keim P, Kelley ST, Knights D, Koester I, Kosciolek T, Kreps J, Langille MGI, Lee J, Ley R, Liu Y-X, Loftfield E, Lozupone C, Maher M, Marotz C, Martin BD, McDonald D, McIver LJ, Melnik AV, Metcalf JL, Morgan SC, Morton JT, Naimey AT, Navas-Molina JA, Nothias LF, Orchanian SB, Pearson T, Peoples SL, Petras D, Preuss ML, Pruesse E, Rasmussen LB, Rivers A, Robeson II MS, Rosenthal P, Segata N, Shaffer M, Shiffer A, Sinha R, Song SJ, Spear JR, Swafford AD, Thompson LR, Torres PJ, Trinh P, Tripathi A, Turnbaugh PJ, Ul-Hasan S, van der Hooft JJJ, Vargas F, Vázquez-Baeza Y, Vogtmann E, von Hippel M, Walters W, Wan Y, Wang M, Warren J, Weber KC, Williamson CHD, Willis AD, Xu ZZ, Zaneveld JR, Zhang Y, Zhu Q, Knight R, Caporaso JG (2019) Reproducible, interactive, scalable and extensible microbiome data science using QIIME 2. Nat Biotechnol 37:852–857. <https://doi.org/10.1038/s41587-019-0209-9>

5.	Callahan BJ, McMurdie PJ, Rosen MJ, Han AW, Johnson AJA, Holmes SP (2016) DADA2: high-resolution sample inference from Illumina amplicon data. Nat Methods 13:581–583. <https://doi.org/10.1038/nmeth.3869>

6.	Rognes T, Flouri T, Nichols B, Quince C, Mahé F (2016) VSEARCH: a versatile open source tool for metagenomics. PeerJ 4:e2584. <https://doi.org/10.7717/peerj.2584>

7.	Katoh K, Standley DM (2013) MAFFT multiple sequence alignment software version 7: improvements in performance and usability. Mol Biol Evol 30:772–780. <https://doi.org/10.1093/molbev/mst010>

8.	Stamatakis A (2014) RAxML version 8: a tool for phylogenetic analysis and post-analysis of large phylogenies. Bioinformatics 30:1312–1313. <https://doi.org/10.1093/bioinformatics/btu033>

9.	Oksanen J, Simpson G, Blanchet F, Kindt R, Legendre P, Minchin P, O’Hara R, Solymos P, Stevens M, Szoecs E, Wagner H, Barbour M, Bedward M, Bolker B, Borcard D, Carvalho G, Chirico M, De Caceres M, Durand S, Evangelista H, FitzJohn R, Friendly M, Furneaux B, Hannigan G, Hill M, Lahti L, McGlinn D, Ouellette M, Ribeiro Cunha E, Smith T, Stier A, Ter Braak C, Weedon J (2024) vegan: community Ecology Package. R package version 2.6-8. https://CRAN.R-project.org/package=vegan

10.	Segata N, Izard J, Waldron L, Gevers D, Miropolsky L, Garrett WS, Huttenhower C (2011) Metagenomic biomarker discovery and explanation. Genome Biol 12:R60. <https://doi.org/10.1186/gb-2011-12-6-r60>

11.	Kolde R (2019) pheatmap: Pretty Heatmaps. R package version 1.0.12. <https://CRAN.R-project.org/package=pheatmap>

12.	De Cáceres MD, Legendre P (2009) Associations between species and groups of sites: indices and statistical inference. Ecology 90:3566–3574. <https://doi.org/10.1890/08-1823.1>

13.	R Core Team R (2013) R: A language and environment for statistical computing

14.	Nguyen NH, Song Z, Bates ST, Branco S, Tedersoo L, Menke J, Schilling JS, Kennedy PG (2016) FUNGuild: an open annotation tool for parsing fungal community datasets by ecological guild. Fungal Ecol 20:241-248. https://doi.org/10.1016/j.funeco.2015.06.006

15.	Kwon SL, Cho M, Lee YM, Lee H, Kim C, Kim G-H, Kim J-J (2022) Diversity of the bambusicolous fungus *Apiospora* in Korea: discovery of new *Apiospora* species. Mycobiology 50:302–316. <https://doi.org/10.1080/12298093.2022.2133808>

16.	Hong J-H, Jang S, Heo YM, Min M, Lee H, Lee YM, Lee H, Kim J-J (2015) Investigation of marine-derived fungal diversity and their exploitable biological activities. Mar Drugs 13:4137–4155. <https://doi.org/10.3390/md13074137>

17.	Williamson B, Tudzynski B, Tudzynski P, Van Kan JAL (2007) *Botrytis cinerea*: the cause of grey mould disease. Mol Plant Pathol 8:561–580. <https://doi.org/10.1111/j.1364-3703.2007.00417.x>

18.	Dean R, Van Kan JAL, Pretorius ZA, Hammond‐Kosack KE, Di Pietro A, Spanu PD, Rudd JJ, Dickman M, Kahmann R, Ellis J, Foster GD (2012) The top 10 fungal pathogens in molecular plant pathology. Mol Plant Pathol 13:414–430. <https://doi.org/10.1111/j.1364-3703.2011.00783.x>

19.	Huang L, He J, Tian C-M, Li D-W (2023) Bambusicolous fungi, diseases, and insect pests of bamboo. In: Forest Microbiology. Elsevier, Amsterdam, pp 415–440. [https://doi.org/10.1016/B978-0-443-18694-3.00006-7](https://doi.org/10.1016/b978-0-443-18694-3.00006-7)

20.	Kohler A, Murat C, Costa M (2011) High quality genomic DNA extraction using CTAB and Qiagen genomic-tip. INRA Nancy Equipe Ecogénomique, Champenoux, France

21.	Sim SB, Corpuz RL, Simmonds TJ, Geib SM (2022) HiFiAdapterFilt, a memory efficient read processing pipeline, prevents occurrence of adapter sequence in PacBio HiFi reads and their negative impacts on genome assembly. BMC Genomics 23:157. <https://doi.org/10.1186/s12864-022-08375-1>

22.	Kolmogorov M, Yuan J, Lin Y, Pevzner PA (2019) Assembly of long, error-prone reads using repeat graphs. Nat Biotechnol 37:540–546. <https://doi.org/10.1038/s41587-019-0072-8>

23.	Chen S, Zhou Y, Chen Y, Gu J (2018) fastp: an ultra-fast all-in-one FASTQ preprocessor. Bioinformatics 34:i884–i890. <https://doi.org/10.1093/bioinformatics/bty560>

24.	Vaser R, Sović I, Nagarajan N, Šikić M (2017) Fast and accurate de novo genome assembly from long uncorrected reads. Genome Res 27:737–746. <https://doi.org/10.1101/gr.214270.116>

25.	Aury J-M, Istace B (2021) Hapo-G, haplotype-aware polishing of genome assemblies with accurate reads. NAR Genom Bioinform 3:lqab034. <https://doi.org/10.1093/nargab/lqab034>

26.	Davey JW, Davis SJ, Mottram JC, Ashton PD (2020) Tapestry: validate and edit small eukaryotic genome assemblies with long reads. bioRxiv:059402. <https://doi.org/10.1101/2020.04.24.059402>

27.	Smit A, Hubley R, Green P (2015). RepeatMasker Open-4.0. http://www.repeatmasker.org

28.	Flynn JM, Hubley R, Goubert C, Rosen J, Clark AG, Feschotte C, Smit AF (2020) RepeatModeler2 for automated genomic discovery of transposable element families. Proc Natl Acad Sci U S A 117:9451–9457. <https://doi.org/10.1073/pnas.1921046117>

29.	Brůna T, Hoff KJ, Lomsadze A, Stanke M, Borodovsky M (2021) BRAKER2: automatic eukaryotic genome annotation with GeneMark-EP+ and AUGUSTUS supported by a protein database. NAR Genom Bioinform 3:lqaa108. <https://doi.org/10.1093/nargab/lqaa108>

30.	Keilwagen J, Hartung F, Grau J (2019) GeMoMa: homology-based gene prediction utilizing intron position conservation and RNA-seq data. In: Methods Mol Biol Kollmar M (ed.). Humana Press, New York 1962:161–177. <https://doi.org/10.1007/978-1-4939-9173-0_9>

31.	Lowe TM, Eddy SR (1997) TRNAscan-SE: a program for improved detection of transfer RNA genes in genomic sequence. Nucleic Acids Res 25:955–964. <https://doi.org/10.1093/nar/25.5.955>

32.	Nawrocki EP, Eddy SR (2013) Infernal 1.1: 100-fold faster RNA homology searches. Bioinformatics 29:2933–2935. <https://doi.org/10.1093/bioinformatics/btt509>

33.	Dainat J, Hereñú D, Pucholt P (2020) AGAT: another Gff Analysis Toolkit to handle annotations in any GTF. GFF format Zenodo. https:///10.5281/zenodo.3552717

34.	Pertea G, Pertea M (2020) GFF Utilities: GffRead and GffCompare. F1000Res 9:ISCB Comm J-304. <https://doi.org/10.12688/f1000research.23297.2>

35.	Steinegger M, Söding J (2017) MMseqs2 enables sensitive protein sequence searching for the analysis of massive data sets. Nat Biotechnol 35:1026–1028. <https://doi.org/10.1038/nbt.3988>

36.	Huerta-Cepas J, Szklarczyk D, Heller D, Hernández-Plaza A, Forslund SK, Cook H, Mende DR, Letunic I, Rattei T, Jensen LJ, von Mering C, Bork P (2019) eggNOG 5.0: a hierarchical, functionally and phylogenetically annotated orthology resource based on 5090 organisms and 2502 viruses. Nucleic Acids Res 47. D309–D314. <https://doi.org/10.1093/nar/gky1085>

37.	Cantalapiedra CP, Hernández-Plaza A, Letunic I, Bork P, Huerta-Cepas J (2021) eggNOG-mapper v2: functional annotation, orthology assignments, and domain prediction at the metagenomic scale. Mol Biol Evol 38:5825–5829. <https://doi.org/10.1093/molbev/msab293>

38.	Zhang H, Yohe T, Huang L, Entwistle S, Wu P, Yang Z, Busk PK, Xu Y, Yin Y (2018) dbCAN2: a meta server for automated carbohydrate-active enzyme annotation. Nucleic Acids Res 46:W95–W101. <https://doi.org/10.1093/nar/gky418>

39.	Zheng J, Ge Q, Yan Y, Zhang X, Huang L, Yin Y (2023) dbCAN3: automated carbohydrate-active enzyme and substrate annotation. Nucleic Acids Res 51:W115–W121. <https://doi.org/10.1093/nar/gkad328>

40.	Hane JK, Paxman J, Jones DAB, Oliver RP, de Wit P (2019) “CATAStrophy,” a genome-informed trophic classification of filamentous plant pathogens–how many different types of filamentous plant pathogens are there? Front Microbiol 10:3088. <https://doi.org/10.3389/fmicb.2019.03088>

40.	Blin K, Shaw S, Kloosterman AM, Charlop-Powers Z, Van Wezel GP, Medema MH, Weber T (2021) antiSMASH 6.0: improving cluster detection and comparison capabilities. Nucleic acids research, 49(W1):W29-W35. https://doi.org/10.1093/nar/gkab335

41.	Jones DA, Rozano L, Debler JW, Mancera RL, Moolhuijzen PM, Hane JK (2021) An automated and combinative method for the predictive ranking of candidate effector proteins of fungal plant pathogens. Sci Rep 11(1):19731. https://doi.org/10.1038/s41598-021-99363-0

42.	Wang H, Marcišauskas S, Sánchez BJ, Domenzain I, Hermansson D, Agren R, Nielsen J, Kerkhoven EJ (2018a) RAVEN 2.0: A versatile toolbox for metabolic network reconstruction and a case study on *Streptomyces coelicolor*. PLOS Comp Biol 14:e1006541. <https://doi.org/10.1371/journal.pcbi.1006541>

43.	Caspi R, Billington R, Keseler IM, Kothari A, Krummenacker M, Midford PE, Ong WK, Paley S, Subhraveti P, Karp PD (2020) The MetaCyc database of metabolic pathways and enzymes-a 2019 update. Nucleic Acids Res 48:D445–D453. <https://doi.org/10.1093/nar/gkz862>

44.	Altschul SF, Gish W, Miller W, Myers EW, Lipman DJ (1990) Basic local alignment search tool. J Mol Biol 215:403–410. [https://doi.org/10.1016/S0022-2836(05)80360-2](https://doi.org/10.1016/s0022-2836(05)80360-2)

45.	Siewers V, Kokkelink L, Smedsgaard J, Tudzynski P (2006) Identification of an abscisic acid gene cluster in the grey mold *Botrytis cinerea*. Appl Environ Microbiol 72:4619–4626. [https://doi.org/10.1128/AEM.02919-05](https://doi.org/10.1128/aem.02919-05)

46.	Kearse M, Moir R, Wilson A, Stones-Havas S, Cheung M, Sturrock S, Buxton S, Cooper A, Markowitz S, Duran C, Thierer T, Ashton B, Meintjes P, Drummond A (2012) Geneious Basic: an integrated and extendable desktop software platform for the organization and analysis of sequence data. Bioinformatics 28(12):1647–1649. https://doi.org/10.1093/bioinformatics/bts199
