# Supplementary material for: Exploring Multifaceted Roles of Bambusicolous Apiospora in Phyllostachys bambusoides
Source: Microb Ecol. 2025 Nov 5;88(1):115. doi: 10.1007/s00248-025-02631-z (PMC12586400; doi:10.1007/s00248-025-02631-z)
Supplement: Supplementary file 1 — (DOCX 8.12 MB) [file 248_2025_2631_MOESM1_ESM.docx]

**
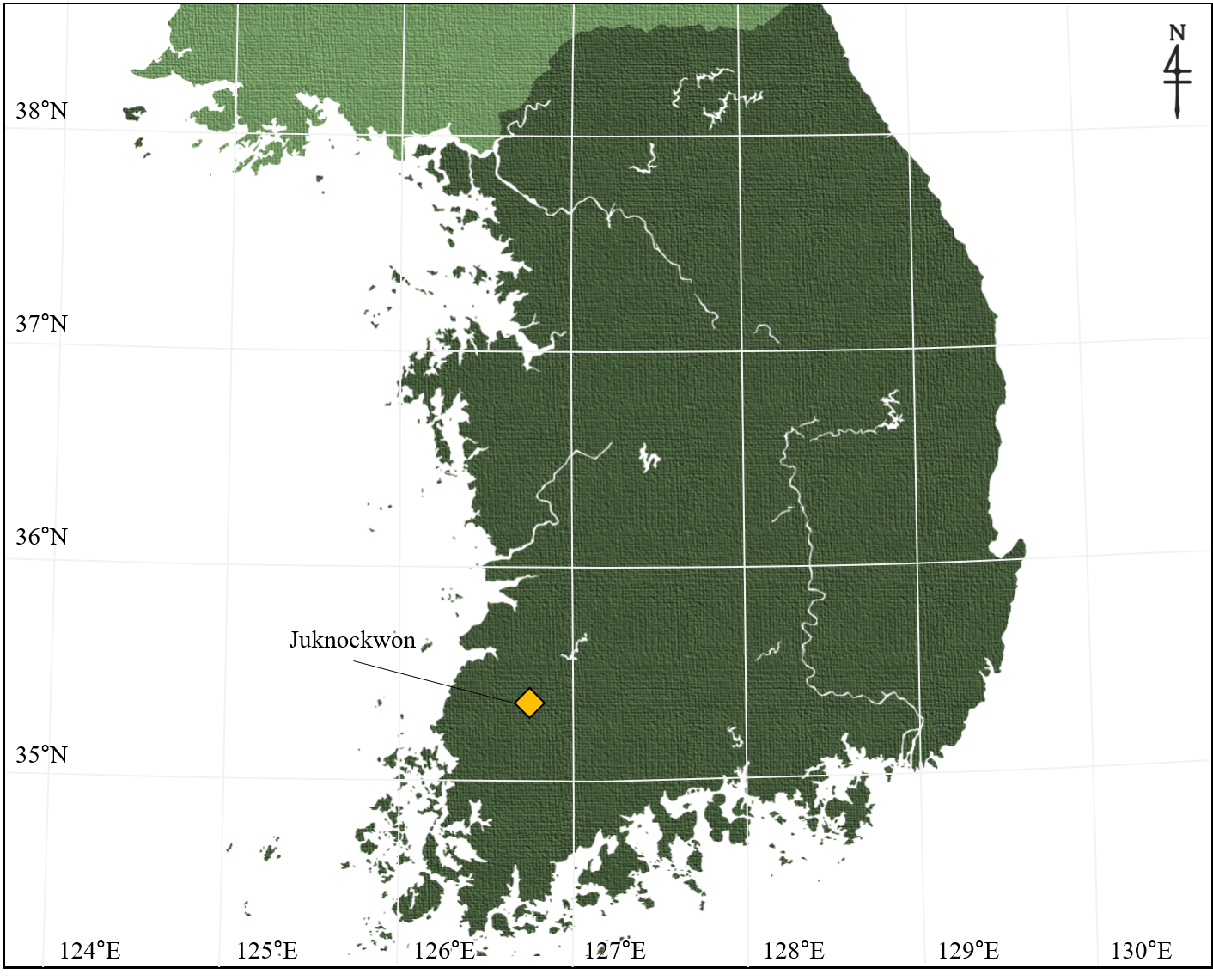
**

**Fig. S1.** Geographic map showing the sampling sites of *Phyllostachys* *bambusoides* forests in Korea.

**
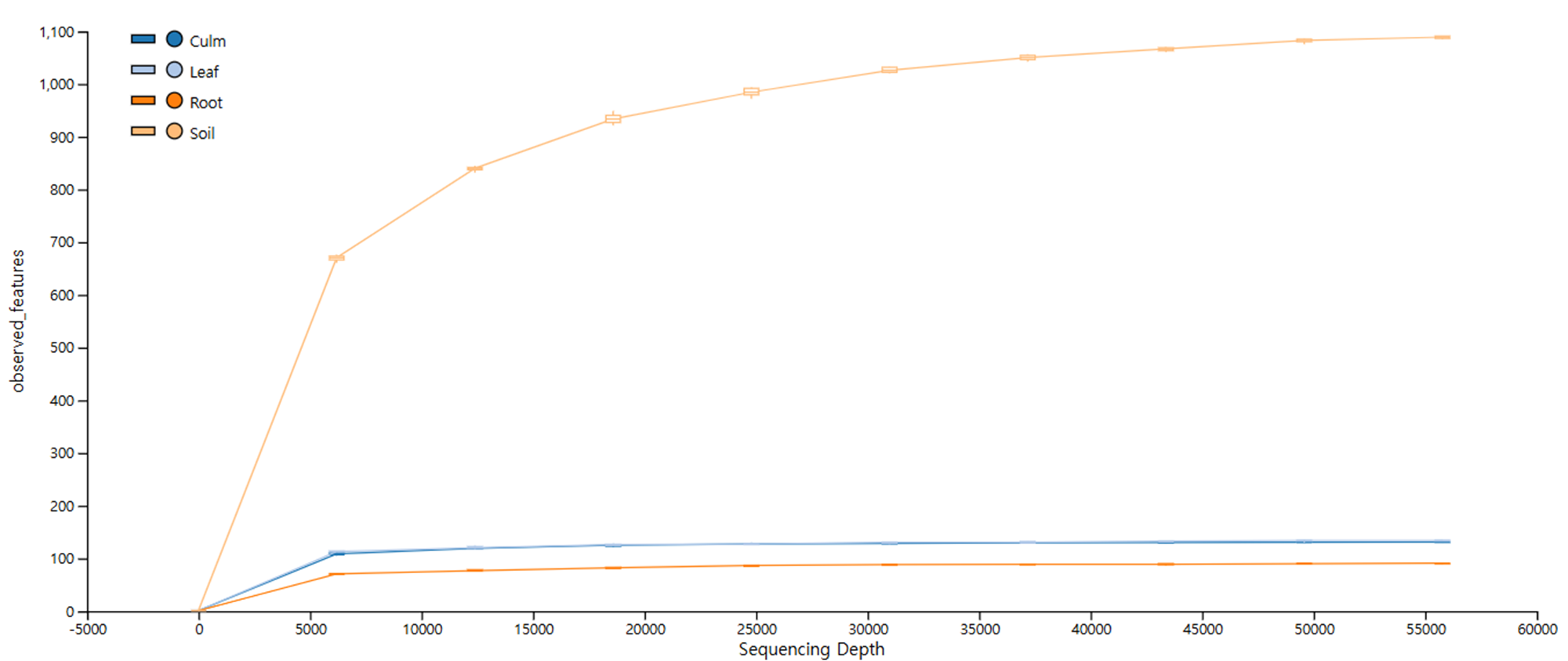
**

**Fig. S2.** Rarefaction curves of the fungal community at different compartment in bamboo forest.


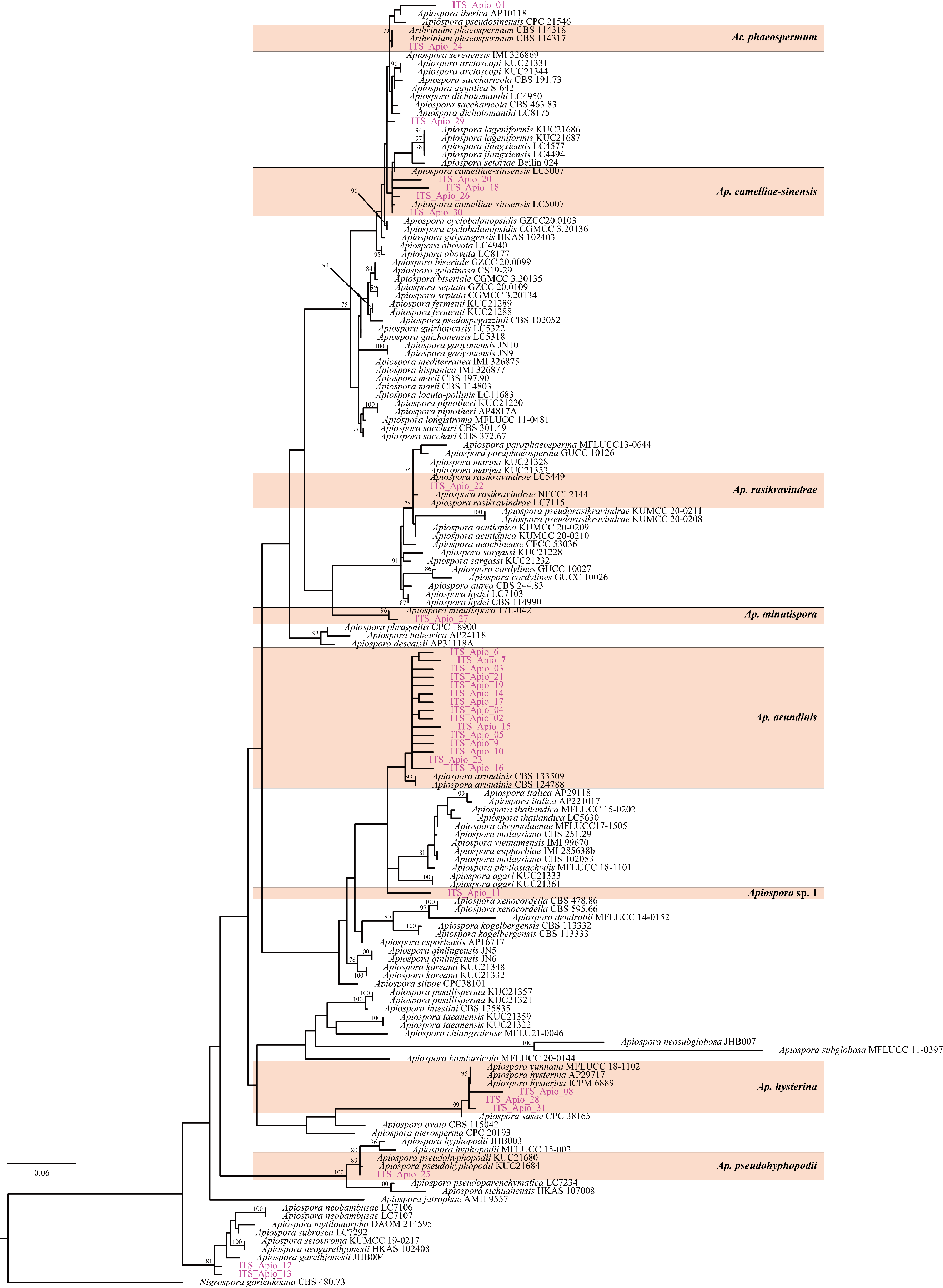


**Fig. S3.** Phylogenetic analysis of bambusicolous endophytic *Apiospora*. ML tree based on ITS datasets. The node numbers indicate the ML bootstrap support (BS) > 70%. *Apiospora* candidates examined in this study are shown in purple color. The boxes indicate the identified *Apiospora* species at the species level.


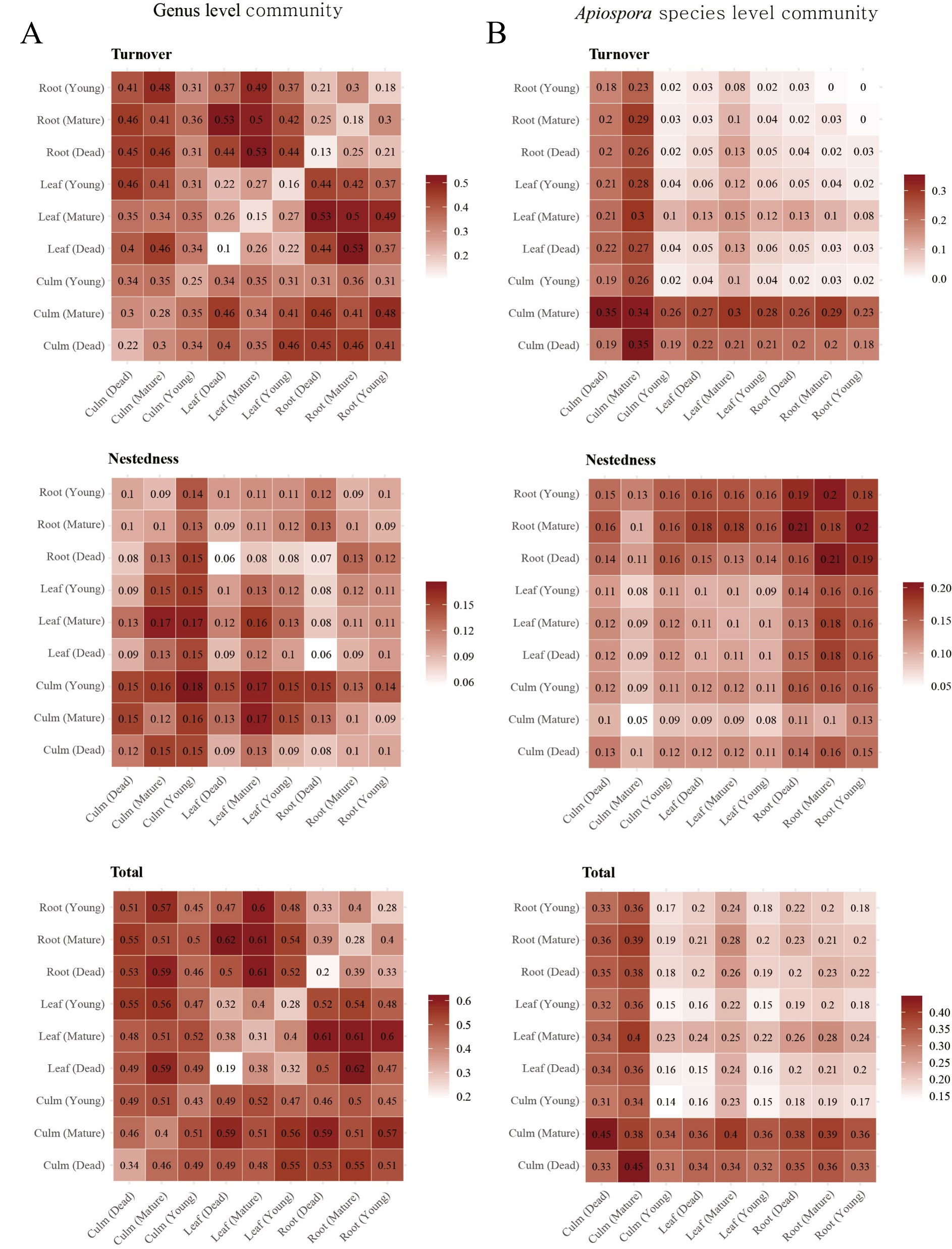


**Fig. S4.** Partitioning of Sørensen-based β-diversity into turnover, nestedness, and total components at the **A** genus level community and **B** *Apiospora* species level community across bamboo tissue types and developmental stages.


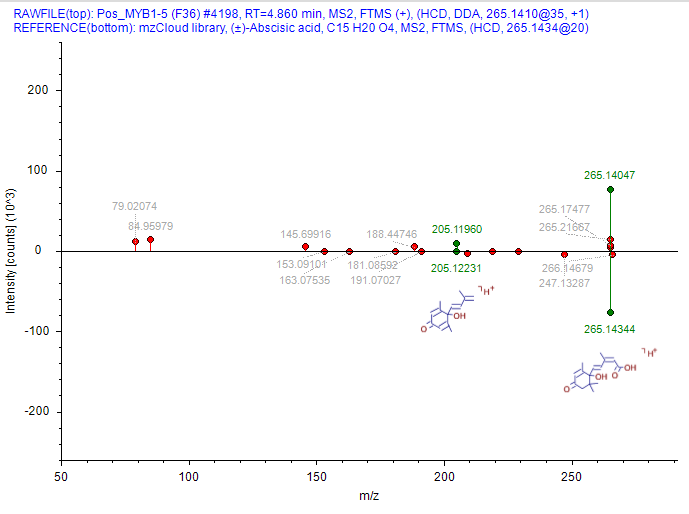


**Fig. S5.** The MS/MS spectral matching of the experimental spectrum and ABA standard spectrum in mzCloud library.

**
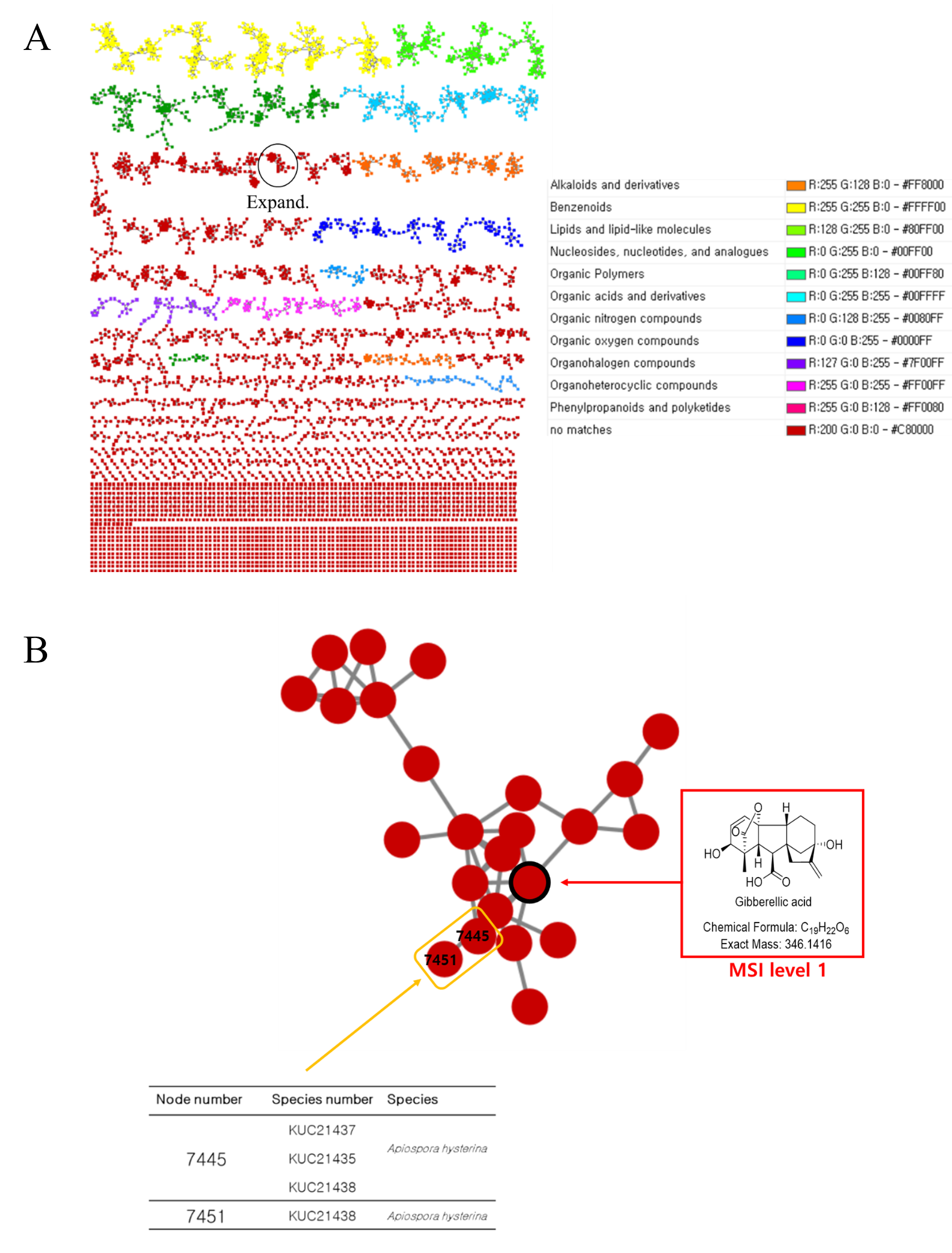
**

**Fig. S6.** Molecular networking of EtOAc extracts of *Apiospora* spp. culture medium. **A** Global molecular network generated from LC–MS/MS data. **B** Expand the specific cluster.

**
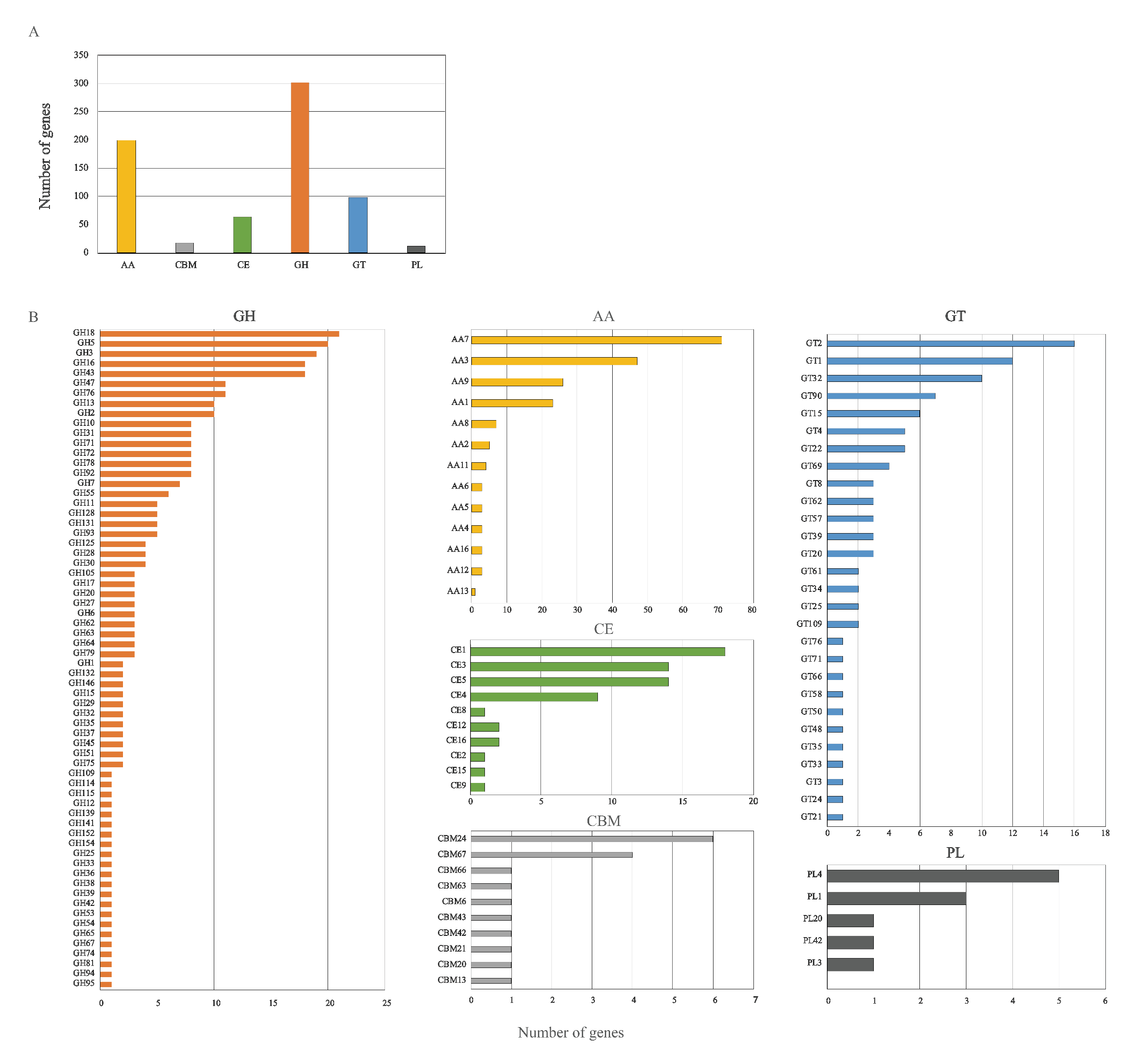
**

**Fig. S7.** Distribution of Carbohydrate-active enzyme (CAZyme). **A** Gene numbers of CAZyme classes: auxiliary activity (AA), carbohydrate-binding modules (CBM), carbohydrate esterase (CE), glycoside hydrolase (GH), glycosyl transferase (GT), and polysaccharide lyase activity (PL); **B** Gene numbers of CAZymes families according to CAZyme classes.





**Fig. S8.** The number of Biosynthetic Gene Clusters (BGCs) of *Ap. hysterina* KUC21437.
